# Effect of aerobic exercise on amyloid accumulation in preclinical Alzheimer’s: A 1-year randomized controlled trial

**DOI:** 10.1371/journal.pone.0244893

**Published:** 2021-01-14

**Authors:** Eric D. Vidoni, Jill K. Morris, Amber Watts, Mark Perry, Jon Clutton, Angela Van Sciver, Ashwini S. Kamat, Jonathan Mahnken, Suzanne L. Hunt, Ryan Townley, Robyn Honea, Ashley R. Shaw, David K. Johnson, James Vacek, Jeffrey M. Burns

**Affiliations:** 1 University of Kansas Alzheimer’s Disease Center, Fairway, KS, United States of America; 2 Department of Psychology, University of Kansas, Lawrence, KS, United States of America; 3 Department of Radiology, University of Kansas Health System, Kansas City, KS, United States of America; 4 Department of Biostatistics and Data Science, University of Kansas Medical Center, Kansas City, KS, United States of America; 5 Department of Neurology, University of California–Davis, Sacramento, CA, United States of America; 6 Department of Cardiovascular Medicine, University of Kansas Health System, Kansas City, KS, United States of America; University of Melbourne, AUSTRALIA

## Abstract

**Background:**

Our goal was to investigate the role of physical exercise to protect brain health as we age, including the potential to mitigate Alzheimer’s-related pathology. We assessed the effect of 52 weeks of a supervised aerobic exercise program on amyloid accumulation, cognitive performance, and brain volume in cognitively normal older adults with elevated and sub-threshold levels of cerebral amyloid as measured by amyloid PET imaging.

**Methods and findings:**

This 52-week randomized controlled trial compared the effects of 150 minutes per week of aerobic exercise vs. education control intervention. A total of 117 underactive older adults (mean age 72.9 [7.7]) without evidence of cognitive impairment, with elevated (n = 79) or subthreshold (n = 38) levels of cerebral amyloid were randomized, and 110 participants completed the study. Exercise was conducted with supervision and monitoring by trained exercise specialists. We conducted 18F-AV45 PET imaging of cerebral amyloid and anatomical MRI for whole brain and hippocampal volume at baseline and Week 52 follow-up to index brain health. Neuropsychological tests were conducted at baseline, Week 26, and Week 52 to assess executive function, verbal memory, and visuospatial cognitive domains. Cardiorespiratory fitness testing was performed at baseline and Week 52 to assess response to exercise. The aerobic exercise group significantly improved cardiorespiratory fitness (11% vs. 1% in the control group) but there were no differences in change measures of amyloid, brain volume, or cognitive performance compared to control.

**Conclusions:**

Aerobic exercise was not associated with reduced amyloid accumulation in cognitively normal older adults with cerebral amyloid. In spite of strong systemic cardiorespiratory effects of the intervention, the observed lack of cognitive or brain structure benefits suggests brain benefits of exercise reported in other studies are likely to be related to non-amyloid effects.

**Trial registration:**

NCT02000583; ClinicalTrials.gov.

## Introduction

There is increasing interest in the role of exercise in the prevention and treatment of Alzheimer’s disease and related cognitive disorders given the growth of the older adult population. Though not all studies agree [1], accumulating evidence suggests that aerobic exercise may protect against cognitive decline and dementia [2–5]. Ongoing work will provide more definitive evidence regarding the cognitive benefits of exercise [6], but aerobic exercise remains among the most promising and cost-effective strategies for delaying or preventing cognitive decline and dementia [7,8].

A wealth of data indicate exercise positively impacts brain health. Higher levels of aerobic fitness are associated with age-related improvements or attenuated decline in brain volume and cognition at both cross-section and over time [3,9–13]. In randomized controlled trials, aerobic exercise promotes brain plasticity, attenuate hippocampal atrophy, or even promotes hippocampal volume increases while improving spatial memory [3,5,14,15].

The effect of aerobic exercise on the pathophysiological markers of AD, beta-amyloid and tau, have been less well explored. Animal studies indicate exercise may reduce amyloid burden and modify AD pathophysiology through direct effects on amyloid precursor protein metabolism [16–18] and indirect effects on neurotrophic factors, neuroinflammation, and oxidative stress [16,17,19–21]. Exercise-induced reductions of amyloid also appear to mediate improvements in cognitive functioning in animals [22–25]. Studies in humans assessing the effect of physical activity on AD pathophysiology are limited. Cross-sectional, observational studies in humans have found that greater amounts of self-reported physical activity (i.e., volitional behavior that is part of daily function) is associated with evidence of lower cerebral amyloid levels among cognitively normal adults [26–32], and those at high genetic risk for AD [26,33–35]. It remains unclear whether the lifestyle behaviors causally influence cerebral amyloid, or vice versa, and whether introducing more physical activity through planned exercise can causally mitigate amyloid pathology.

The advent of amyloid imaging creates an opportunity for identifying individuals in the presumptive pre-symptomatic phase of AD, when interventions may have the greatest impact [36]. Approximately 30% of cognitively normal older adults have asymptomatic cerebral amyloidosis and thus meet the NIA and Alzheimer’s Association research criteria for “preclinical AD”, defined as having a cerebral to cerebellar amyloid ratio above a certain, method-dependent threshold. The concept of preclinical AD posits that cerebral amyloid deposition in cognitively normal adults represents a pre-symptomatic stage of AD and individuals with preclinical AD currently represent the earliest feasible stage for trials of AD prevention. Individuals with subthreshold levels of cerebral amyloid (individuals with non-elevated amyloid PET but with quantitative measures near the threshold for being elevated) may be more likely to accumulate clinically significant levels of amyloid and have memory decline [37], suggesting they are good candidates for prevention studies [38].

Our study examined the effects of a 52-week aerobic exercise program on AD pathophysiology (amyloid burden), associated “downstream” neurodegeneration (whole brain and hippocampal volume change) and cognitive decline in cognitively normal individuals with either preclinical AD or with subthreshold levels of cerebral amyloid. We hypothesized that 52 weeks of aerobic exercise would be associated with reduced amyloid accumulation, reduced hippocampal atrophy, and improved performance on a cognitive test battery.

## Materials and methods

### Study design

The Alzheimer’s Prevention through Exercise study (APEx: ClinicalTrials.gov, NCT02000583; trial active between 11/1/2013–11/6/2019) was a 52-week study of aerobic exercise in individuals 65 years and older without cognitive impairment. Based on public health recommendations and our prior work [4,39], we randomized individuals to either 150 minutes per week of supported moderate intensity aerobic exercise or standard of care education control in a 2:1 ratio. The unbalanced design was intended to maximize recruitment and retention with minimal impact in power. Cerebral amyloid load, neurodegeneration, cognition, and cardiorespiratory fitness were measured at baseline and post-intervention. Cognition was also measured at the midpoint of the study. The University of Kansas Medical Center Human Subjects committee approved the protocol (HSC#13376) and written informed consent was obtained from all participants.

### Participants

Participants were recruited as a convenience sample of volunteers through print and online advertising, community talks, and existing databases of individuals willing to be in research studies [40]. Enrollment occurred between March 1, 2014 and October 31, 2018. Interested individuals first underwent a telephone screen of medical history for key inclusion and exclusion criteria including: age of 65 years and older, sedentary or underactive as defined by the Telephone Assessment of Physical Activity [41], on stable medications for at least 30 days, willingness to conduct prescribed exercise (or not) for 52 weeks at a community fitness center, and willingness to undergo an 18F-AV45 PET scan for cerebral amyloid load and learn their individual result (elevated vs non-elevated). Amyloid status was disclosed to all participants regardless of screening status [42]. In-person screening included a clinical assessment by clinician of the University of Kansas Alzheimer’s Disease Center including a Clinical Dementia Rating and Uniform Data Set neuropsychiatric battery [43,44]. Participants could not be insulin-dependent, have significant hearing or vision problems, clinically evident stroke, cancer in the previous 5 years (except for localized skin or cervical carcinomas or prostate cancer), uncontrolled hypertension, or have had recent history (<2 years) of major cardiorespiratory, musculoskeletal or neuropsychiatric impairment, and had to be able to complete graded maximal exercise testing with a respiratory exchange ratio > = 1.0.

We enrolled only those participants who met criteria for elevated cerebral amyloid (see below) as previously described [42,45], until March 2016 when we revised the protocol to allow individuals with subthreshold amyloid levels (cerebral-to-cerebellar standard uptake value ratio (SUVR) threshold > 1.0). This was motivated by recruitment challenges for the preclinical AD group and new evidence that this group accumulates amyloid and is more likely to have associated memory decline [37] and thus may represent an excellent target for early prevention studies.

### Amyloid screening

Florbetapir PET scans were obtained approximately 50 minutes after administration of intravenous florbetapir 18F-AV45 (370 MBq) on a GE Discovery ST-16 PET/CT scanner. Two PET brain frames of five minutes in duration were acquired continuously, summed, and attenuation corrected. To determine amyloid status three experienced raters interpreted all images independently and without reference to any clinical information, as previously described [45]. Raters followed a process that combined both visual and quantitative information to determine status as “elevated” vs “non-elevated.” Final status was determined by majority of the three raters [46,47]. Images were viewed and analyzed using the MIMneuro Amyloid Workflow (version 6.8.7, MIM Software Inc., Cleveland, OH, USA), using florbetapir templates as the target for a two-phase registration: first rigid registration, then deformable registration to a common template space. Raters first reviewed raw PET images visually then examined the cerebellum normalized SUVRs in 6 cortical regions (anterior cingulate, posterior cingulate, precuneus, inferior medial frontal, lateral temporal, and superior parietal cortex) and projection maps comparing SUVRs to an atlas of amyloid negative scans [46]. Participants were eligible for the study if they had an elevated scan or (after March 2016) were in the subthreshold range. We defined subthreshold as a mean cortical SUVR for the 6 ROIs > 1.0, which represented the upper half of non-elevated scans (mean cortical SUVR for non-elevated scans [n = 166] 0.99 [0.06 SD]). Enrolled participants were re-scanned after 52 weeks of intervention.

### Allocation

A study statistician constructed an allocation schedule that was applied by study staff after baseline testing was completed. The study statistician used random number generator to generate blocks of nine in a 2:1 ratio to protect against imbalance if recruitment fell short Participants were prospectively assigned to treatment versus control from this schedule using REDCap’s randomization module which restricts access and viewing once uploaded.

### Intervention

Participants in the education control group were provided with standard exercise public health information and received a membership to a community exercise facility after completion of the study.

For those randomized to the aerobic exercise group, the intervention was conducted at their nearest study-certified exercise facility under the guidance of certified personal trainers employed by the community exercise facility. They were asked to refrain from changing their regular physical activities other than those prescribed by the study team. Methods for ensuring study protocol compliance and ongoing training refreshers have been published previously [4,48,49]. Personal trainers oversaw prescription for weekly exercise duration and intensity under the direction of the study team. At each session, participants manually recorded the duration of exercise on an exercise study log. Exercise began with a goal of 60 total minutes during Week 1 and increased by approximately 21 min/week until achieving 150 min/week of aerobic exercise. Participants exercised 3–5 days a week, never more than 50 minutes a day to reduce the likelihood of overuse injury. Intensity was prescribed as a target heart rate zone (F4 or FT4, Polar Electro Inc., Lake Success, NY) based on the percentage of heart rate reserve (HRR) as calculated by the Karvonen formula. Beginning at 40–55% of HRR (% of the difference between maximal and resting), target heart rate zones were increased by 10% of HRR every 3 months.

Trainers supervised all exercise sessions for the first 6 weeks of exercise and at least once weekly thereafter. Treadmill walking served as the primary exercise mode but participants were allowed to use a different aerobic modality if requested to alleviate boredom or accommodate discomfort. No compensation was provided to participants beyond the fees paid to the exercise facility for memberships and trainer time. We have previously demonstrated that our methods using community fitness facilities and trainers can deliver a well-controlled exercise dose with rigor and a high level of adherence, comparable to lab-based methods [4,49].

### Adherence and safety

Trainers asked about changes in health status (adverse events [AE]) at every visit. Study staff inquired about AEs and medication changes during scheduled telephone check-ins every 6 weeks, or during incidental contact at weekly exercise facility visits. An independent safety committee reviewed AEs quarterly. Intent-to-treat analyses were performed on all enrollees (n = 117). We separately assessed individuals who participated in the trial per-protocol (n = 92) by complying with at least 80% of the intervention exercise prescription [4].

### Outcomes

This study sought to provide evidence of a specific effect on AD pathophysiology (i.e., disease-modifying effect) of aerobic exercise on AD-related pathophysiological change in preclinical AD. We specified our primary outcome as mean change from baseline to 52 weeks in 18F-AV45 standard uptake value ratio (SUVR) with secondary outcomes of MRI measures of change in whole brain and hippocampal volume and cognitive performance measures. To assess the physiologic impact of the intervention, we measured the highest achieved oxygen consumption rate (VO_2_ peak, mL·kg^-1^·min^-1^) during a graded exercise test [4].

Substantial evidence exists demonstrating that aerobic exercise has a preferential effect on cognition, particularly in executive functioning [3,50]. Thus, our cognitive outcome measure of interest was executive function. We also planned to assess key cognitive domains that are associated with asymptomatic cerebral amyloid deposition such as episodic memory and visuospatial function which has been previously associated with aerobic exercise [4]. Raters involved with key outcomes (psychometrician, imaging technicians, exercise physiologists) were blinded to the participant’s intervention group (aerobic exercise or control) and had no interaction with participants beyond the testing visits.

#### Magnetic Resonance Imaging (MRI) of brain anatomy

MRI of the brain was performed at baseline and 52-week follow up testing in a Siemens 3.0 Tesla Skyra scanner. We obtained a high-resolution T1 weighted image (MP-RAGE; 1x1x1.2mm voxels; TR = 2300ms, TE = 2.98ms, TI = 900ms, FOV 256mmx256mm, 9°flip angle) for detailed anatomical assessment. We used the Freesurfer image analysis suite (ver. 5.2 http://surfer.nmr.mgh.harvard.edu/) for volumetric segmentation optimized for longitudinal data [51], extracting hippocampal and total gray matter volume change as measures of neurodegeneration.

#### Cognitive test battery

A trained psychometrist performed a comprehensive cognitive test battery at baseline and again at Week 26 and Week 52, employing validated, alternate versions of tests every other visit. We created composite scores for three cognitive domains (executive function, verbal memory, visuospatial processing) using Confirmatory Factor Analysis (CFA) in MPlus software. We standardized scores to baseline mean and standard deviation, thus scores at Week 26 and Week 52 can be interpreted as a change from baseline. The executive function domain composite score was made up of verbal fluency (the sum of animals and vegetables) [52], Trailmaking Test B [53], Digit Symbol Substitution test [54], and the interference portion of the Stroop test [55]. The verbal memory domain composite score was made up of the immediate and delayed portions of the Logical Memory Test [54], and the sum of free recall trials of the Selective Reminding Test [56]. The visuospatial domain composite score was made up of scores from Block Design [54], space relations, the paper folding test, hidden pictures, and identical pictures [57]. We included the combined cognitive scores as outcomes in subsequent models. Missing data were accounted for using full information maximum likelihood algorithm. To evaluate model fit, we used Root Mean Squared Error of Approximation (RMSEA), a measure of the discrepancy between predicted and observed model values. Values closer to 0 indicate better fit (preferred values are <0.09). We report a comparative fit index (CFI) that estimates the relative fit of a model compared to an alternative model, in which a CFI >0.90 indicates good fit. Typically, these multiple fit indices are considered together, as opposed to relying on any one indicator.

#### Graded maximal exercise test

We assessed cardiorespiratory fitness at baseline and Week 52 as the highest oxygen consumption attained (VO_2_ peak) during cardiorespiratory exercise testing on a treadmill to maximal capacity or volitional termination [4].

#### Genotyping

APOE genotype determination Whole blood was collected and stored at -80C until genetic analyses could be conducted. To determine APOE genotype, frozen whole blood was assessed using a Taqman single nucleotide polymorphism (SNP) allelic discrimination assay (ThermoFisher). APOE4, APOE3, and APOE2 alleles were distinguished using Taqman probes to the two APOE-defining SNPs, rs429358 (C_3084793_20) and rs7412 (C_904973_10). The term “APOE4 carrier” was used to describe the presence of 1 or 2 APOE4 alleles.

### Statistical analysis

Descriptive statistics were generated, including means, standard deviations and ranges for continuous measures, and frequencies and relative frequencies for categorical measures. For primary study endpoints with baseline and 12-month follow-up data only, we calculated differences between pre- and post-treatment measures and compared these differences with two-sample t-tests for intent-to-treat analyses. For further assessment among the per protocol population we used covariate adjustment by analyzing these difference scores as a function of the treatment group and other covariates (age, sex, education, and PET amyloid status [elevated vs. subthreshold]) using ordinary least squares regression. For cognition endpoints measured at three time points (baseline, 26-, and 52-weeks), linear mixed models were used. We used a random intercept for subject to account for repeated measures, and treated time as a linear explanatory variable. Unadjusted analyses included treatment group, time, and their interaction, with the interaction test term providing the test for interaction effect using a t-test of that parameter from the model for intent-to-treat results. This approach also allowed for further covariate adjustment for sex, age, education, and PET amyloid status among the per-protocol subgroup.

All statistical methods assessed appropriate model assumptions. For continuous measures, this involved residual analyses to assess normality and variance homogeneity assumptions.

At the time of study design, no previous exercise studies in humans had measured in vivo amyloid. Therefore, we powered our primary outcome from prior investigational compound work. A 78-week study assessing Bapineuzumab in AD reported an effect size of d = 1.98 [58]. Given the differences in our proposed study (preclinical AD sample, lower expected amyloid burden, shorter duration and expectation of a lower exercise effect) we estimated effect size of only 40% as large, (resultant d = 0.79), yielding 93% power to detect this conservative anticipated effect of exercise on amyloid burden. Our initial enrollment goal was 100. Subsequent to including individuals with subthreshold amyloid, we increase our enrollment goal to 120.

Data were captured using REDCap [2] which allowed for secure randomization and role based access to data capture forms. The analysis for this project was generated using SAS software, Version 9.4 for Windows (SAS Institute Inc., Cary, NC, USA).

## Results

### Participants

**Fig 1:**
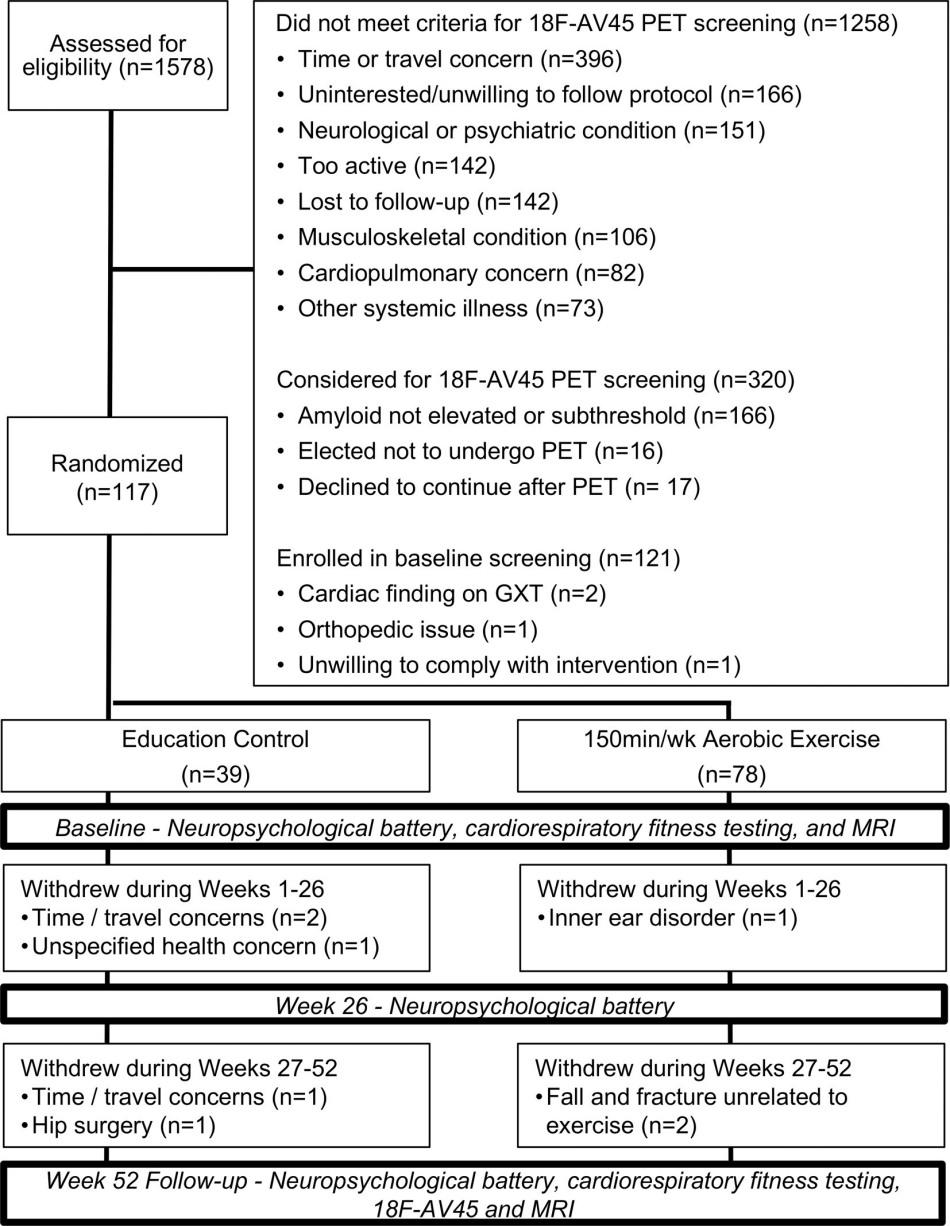
APEx study CONSORT diagram.

A total of 1578 individuals were assessed for study eligibility from November 2013 to October 2018. The flow of participants from screening through study completion is shown in Fig 1. Participants (n = 117) were randomized to either the aerobic exercise (n = 78) or control (n = 39) intervention groups.

A total of 109 participants (93%: control n = 34, aerobic exercise n = 75) completed the study. There were no significant differences across intervention groups in demographic and baseline characteristics (p>0.05, Table 1).

**Table 1:** Enrolled participant demographics.

Measure	Education Control (n = 39)	Aerobic Exercise (n = 78)	p-value
Age (y)	72.2 (5.3)	71.2 (4.8)	0.31
Sex, female n(%)	24 (61.5)	55 (70.5)	0.44
Education (y)	16.2 (2.2)	16.1 (2.4)	0.71
APOE ε4 carriers n(%)*	15 (38.5)	36 (46.2)	0.51
Race/Ethnicity: White, not Latino n(%)	35 (89.7)	77 (98.7)	0.08
African American n(%)	4 (10.3)	1 (1.3)	
Baseline MMSE (mean, range)	29.1 (26–30)	29.1 (26–30)	0.51
Elevated amyloid status: n(%)	27 (69.2)	52 (66.7)	0.94
subthreshold n(%)	12 (30.8)	26 (33.3)	

Mean (standard deviation), unless otherwise noted.

*Four individuals declined genotyping. APOE = Apolipoprotein ε4 genotype; MMSE = Mini-Mental State Exam.

### Adherence to exercise protocol

The aerobic exercise group completed an average of 84.6% (SD 25.8%) minutes of the prescribed exercise dose. The control group did not report weekly exercise. However, the control group remained underactive or sedentary during the intervention as evidence by their self-report of weekly physical activity [59]. The control group reported a -778 calorie (SD 5101) reduction in moderate intensity activity from baseline to Week 52. In contrast, the aerobic exercise group increased moderate intensity physical activity by 1853 calories (SD 5019).

### Outcomes of interest

We provide pre-specified analyses for both the intent-to-treat cohort of 117 enrollees and a per-protocol cohort of 92 individuals who were protocol adherent (as defined as achieving > = 80% of prescribed exercise minutes).

Primary and secondary outcomes are detailed in Table 2. In the intent-to-treat group, there was a strong physiologic effect of aerobic exercise on cardiorespiratory fitness, with the aerobic exercise group increasing VO_2_ peak by 11% compared to 1% in the control group. There was no apparent effect of intervention on the primary outcome measure of change in global cerebral amyloid (p>0.9). Aerobic exercise was not associated with change in executive function, verbal memory, or visuospatial function (p> = 0.3). Aerobic exercise was not associated with a change in whole brain or hippocampal volume (p>0.1). These results were unchanged when we excluded the subthreshold amyloid group and assessed only those with elevated amyloid (n = 79; S1 Table).

**Table 2:** Primary outcome measures in the Intent-to-Treat group.

Outcome measures	Timepoint	Education (n = 39^)	Aerobic Exercise (n = 78^)	p-value*
Global Amyloid Burden (SUVR)	Baseline	1.2 (0.2)	1.22 (0.2)	0.93
	Week 52	1.21 (0.2)	1.22 (0.2)	
	Change	0.01 (0.04)	0.01 (0.06)	
VO_2_ peak (mL·kg^-1^·min^-1^)	Baseline	22.7 (5.3)	21.9 (5.2)	0.01
	Week 52	23.0 (4.9)	24.3 (5.8)	
	Change	0.1 (2.5)	2.0 (2.5)	
Whole Brain Volume (mL)	Baseline	1061.7 (114.4)	1068.7 (109.7)	0.12
	Week 52	1059.1 (115.1)	1063.4 (109.1)	
	Change	-2.6 (-7.2)	-5.3 (-8.7)	
Hippocampal Volume (mL)	Baseline	7.6 (1.0)	7.5 (0.8)	0.42
	Week 52	7.6 (1.0)	7.4 (0.8)	
	Change	-0.09 (0.14)	-0.07 (0.10)	
Executive Function Composite	Baseline	-0.042 (0.365)	0.029 (0.458)	0.83
	Week 26	-0.035 (0.389)	0.017 (0.625)	
	Week 52	-0.037 (0.452)	0.018 (0.615)	
Verbal Memory Composite	Baseline	0.032 (0.822)	-0.016 (0.882)	0.69
	Week 26	-0.007 (1.014)	0.003 (0.989)	
	Week 52	0.051 (0.939)	-0.025 (0.935)	
Visuospatial Composite	Baseline	-0.062 (0.572)	0.031 (0.646)	0.30
	Week 26	0.012 (0.659)	-0.006 (0.715)	
	Week 52	0.003 (0.559)	-0.001 (0.643)	

Mean (standard deviation). Cognitive composites at Week 26 and Week 52 can be interpreted as change from baseline.

*2 sample paired t-test comparing baseline and week 52 for amyloid, fitness and volume measures. For cognitive measures, a p-value for treatment by time interaction test from linear mixed models is given.

^Sample size for change in amyloid is Educ:35/Exercise:74. Sample sizes for change in fitness and volumes are Educ:34/Exercise:70 Sample sizes for cognitive measures at baseline, week 26, and week 52 are Educ:39,37,36/Exercise:78,75,75. SUVR = Standard uptake value ratio; VO_2_ peak = peak oxygen consumption during the graded exercise test.

In the per-protocol subset (Table 3; n = 92), there remained a strong physiologic effect of aerobic exercise, with the aerobic exercise group increasing VO_2_ peak by 12.8%. Again, there was no apparent effect of intervention on primary outcome measure of change in global amyloid burden (p>0.7). Aerobic exercise was not associated with change in executive function, verbal memory or visuospatial function (p>0.2). Aerobic exercise was not associated with a change in whole brain or hippocampal volume (p>0.1). When we examined only those participants with elevated amyloid (n = 65) there were no differences in the results (S1 Table).

**Table 3:** Primary outcome measures in the Per-Protocol group.

Outcome measure	Timepoint	Education (n = 39^)	Aerobic Exercise (n = 53^)	p-value*
Global Amyloid Burden (SUVR)	Baseline	1.20 (0.2)	1.23 (0.2)	0.73
	Week 52	1.21 (0.2)	1.24 (0.2)	
	Change	0.01 (0.04)	0.01 (0.06)	
VO_2_ peak (mL·kg^-1^·min^-1^)	Baseline	22.7 (5.3)	22.7 (5.4)	<0.01
	Week 52	23.0 (4.9)	25.2 (5.8)	
	Change	0.1 (2.5)	2.5 (2.4)	
Whole Brain Volume (mL)	Baseline	1061.7 (114.4)	1073.6 (109.3)	0.12
	Week 52	1059.1 (115.1)	1068.0 (108.3)	
	Change	-2.6 (7.2)	-5.5 (7.6)	
Hippocampal Volume (mL)	Baseline	7.6 (1.0)	7.6 (0.7)	0.31
	Week 52	7.6 (1.0)	7.5 (0.7)	
	Change	-0.09 (0.14)	-0.06 (0.10)	
Executive Function Composite	Baseline	-0.042 (0.365)	0.034 (0.392)	0.90
	Week 26	-0.035 (0.389)	0.041 (0.606)	
	Week 52	-0.037 (0.452)	0.012 (0.607)	
Verbal Memory Composite	Baseline	0.032 (0.822)	0.024 (0.861)	0.47
	Week 26	-0.007 (1.014)	-0.039 (0.931)	
	Week 52	0.051 (0.939)	-0.053 (0.839)	
Visuospatial Composite	Baseline	-0.062 (0.572)	0.067 (0.657)	0.22
	Week 26	0.012 (0.659)	0.036 (0.727)	
	Week 52	0.003 (0.559)	0.017 (0.669)	

Mean (standard deviation). Cognitive composites at Week 26 and Week 52 can be interpreted as change from baseline.

* For amyloid, fitness and brain volume measures, a p-value from ordinary least squares regression adjusted for sex, age, education, and amyloid status comparing the change (baseline to week 52) between the two groups is given. For cognitive measures, a p-value for treatment by time interaction test from linear mixed models adjusted for sex, age, education, and amyloid status among per protocol subgroup is given.

^Sample size for amyloid at baseline and week 52 are Educ:39,35/Exercise:53,53. Sample size for VO_2_ at baseline and week 52 are Educ:39,34/Exercise:53,53. Sample size for brain volumes at baseline, week 52 are Educ:34,34/Exercise:52,52. Sample sizes for cognitive measures at baseline, week 26, and week 52 are Educ:39,37,36 /Exercise:53,53,53. SUVR = Standard uptake value ratio; VO_2_ peak = peak oxygen consumption during the graded exercise test.

There were 122 adverse events. Three incidental cardiac findings were discovered at baseline exercise testing and one fall at home during screening for which participants did not receive clearance to continue participation, leaving 118 adverse events following randomization. The education control group had 118 adverse events: 10 mild, 3 moderate and 5 severe, all unrelated to the intervention. The aerobic exercise group had 31 mild (e.g., joint pain resolving with exercise modification), 2 moderate (e.g. joint pain temporarily halting exercise), and 0 severe event related to the intervention, and 48 mild, 12, moderate, and 7 severe events unrelated to the intervention. Examples of mild severity events included seasonal allergies and joint pain resolving with exercise modification. Examples of moderate severity events included outpatient eye surgery and joint pain altering exercise. Examples of severe events included falls at home and hospitalization for gastrointestinal infection. The Data and Safety Monitoring Committee (DSMC) was comprised of 3 physicians unaffiliated with the authors. Adverse events were submitted for review to the DSMC quarterly or within 48 hours if a serious adverse event. Adverse events are summarized in S2 Table.

## Discussion

This is the one of the first randomized controlled trials to prospectively assess the effect of aerobic exercise on cerebral beta-amyloid accumulation in humans. We found no evidence that one year of aerobic exercise influences cerebral amyloid burden in a cohort of cognitively normal participants with elevated and subthreshold levels of amyloid, individuals who are at highest risk of clinically significant amyloid accumulation. We did find significant and meaningful changes in cardiorespiratory fitness suggesting the intervention was of sufficient intensity and duration to provoke physiologic effects. Despite this, however, we did not find aerobic exercise effects on whole brain volume, hippocampal volume, or cognitive measures. Our observed atrophy rates were consistent with those previously reported in cognitively normal older adults [60]. We believe these null findings support a hypothesis that the widely reported brain benefits of exercise are modest and driven mechanistically by the mitigation of non-amyloid pathologies.

It is important to consider the context of our findings in a highly selected sample that likely skewed towards fewer age-related pathologies, such as subclinical cerebrovascular disease, than most studies in the literature. We assessed over 1,500 participants for eligibility (see Fig 1) and excluded those with cardiopulmonary concerns and systemic illnesses while retaining those (largely through participant self-selection) interested in potentially participating in a year of rigorous exercise. Importantly, we performed careful clinical and cognitive assessments to exclude those with cognitive impairment, despite the presence of cerebral amyloid; thus this group is likely enriched with unmeasured (and currently poorly defined) resilience factors, such as the absence of cerebrovascular disease or other age related pathologies. The lack of observed exercise effects on amyloid, cognitive, or brain structure outcomes despite clear exercise related effects on physiologic outcomes (cardiorespiratory fitness) leads us to hypothesize that the brain benefits of aerobic exercise observed widely in the literature are not driven by effects on AD pathology but instead are likely driven by the mitigation of aging related vascular or other non-amyloid pathologies. Indeed, recent work has identified cerebrovascular outcomes and important mediators of cognitive change following exercise [5,14,61].

It remains possible that our results are related to Type II error where a true effect is obscured by lack of power or methodological issues. Our null finding for an effect of aerobic exercise on amyloid accumulation is surprising given the number of animal exercise studies reporting reduced amyloid accumulation rates and lower amyloid loads [16,17,19–21]. However, small human intervention studies have examined the impact of exercise on amyloid with inconclusive results. At least three intervention studies have examined the impact of exercise on serum amyloid concentration, with none reporting reliable reductions in amyloid as a consequence of exercise [62–64]. The amyloid tracer we employed (18F-AV45) may lack sufficient sensitivity/specificity to index subtle changes in amyloid induced by exercise in cognitively normal older adults. In the overall group (n = 106) we observed a 0.8% (SD 4.4%) increase in amyloid compared to reported annual changes of 1–4%, a range influenced by where an individual is on the sigmoid curve of accumulation over the lifespan [65]. Additionally, when examining our subgroups, the elevated group had a 1.5% (SD 4.5%) annual rate of accumulation compared to a decline of -0.9% (SD 3.6%) in subthreshold group, a decline that was not in line with our expectations for this group. Recent serial amyloid PET studies suggest that reference region selection (i.e., whole cerebellum vs cerebellar white matter) can influence measured change over time and that annual participant scan variance may be higher than the expected annual rate of amyloid PET change, especially when only two data points are present [66]. However, the tracer can sufficiently track dose-related amyloid change in investigational medication trials [67], suggesting that if our failure to observe changes was related to measurement error, exercise is unlikely to have a large effect on cerebral amyloid levels.

It is possible that our inclusion of individuals in the subthreshold range (n = 38) who were not elevated reduced our ability to detect reductions in amyloid by enhancing a floor effect. However, when assessing only those in the elevated group (n = 79), there were no trends suggesting an effect of aerobic exercise on amyloid accumulation. Additionally, the potential benefits of aerobic exercise to influence cerebral amyloid may require a longer duration than 52 weeks. One year may not be long enough to meaningfully alter amyloid levels or the rate of accumulation. Future studies looking at more than two amyloid PET time points to reduce scan to scan variance and longer time interval (at least 2 years) may be important to investigate whether exercise can impact the rate of amyloid accumulation. The non-significantly higher proportion of E4 carriers in the treatment group may have subtly impacted cognitive decline and amyloid accumulation, potentially obscuring our ability to detect a benefit of the intervention. As a sensitivity analysis, we also tested our models with SUVR as a covariate, and with APOE4 and APOE4 by Treatment Arm as factors. There was no appreciable change in our results in these analyses (data not shown). Though we detected no difference in carrier versus non-carrier performance, brain volume change, or amyloid accumulation, over 1-year, future studies may wish to consider E4 carriage as a blocking variable for randomization

Our lack of effect on our secondary outcomes of brain volume and cognitive performance was surprising, especially given the strong physiologic effects of the exercise intervention on cardiorespiratory fitness. Practice effects, especially in cognitively normal older adults, reduce power to discern group differences in cognitive performance [68,69] but despite this, a number of well-designed RCTs have shown that aerobic exercise benefits cognition in older adults, though not specifically in those with elevated cerebral amyloid [2–5,50]. Many studies have also demonstrated benefits to whole brain gray matter and hippocampal volume, with one notable study reporting a decrease in whole brain gray matter volume after 12 months of resistance training [70]. We have previously suggested that cardiorespiratory fitness gains are critical for cognitive or brain improvements [4,49,71]. Simply exercising without increasing cardiorespiratory fitness, and therefore eliciting associated physiological and biochemical adaptations, does not appear to support brain or cognitive changes. Despite significantly increasing maximal cardiorespiratory capacity in this trial, we did not identify the same relationship in the present study. This may suggest that those with elevated amyloid are more resistant to the putative brain benefits of aerobic exercise.

There are several additional limitations to this study. Our sample was almost exclusively White, non-Hispanic and highly educated. This severely limits the generalizability of our findings and highlights structural racism and inequity related to clinical trial access. As a result, we have begun assessing the design of our trials and increased our efforts to inclusively design our exercise trials with and for underrepresented communities [72–75]. An additional limitation is the use of self-reported physical activity versus an activity monitor and lack of a treatment fidelity analysis. It is possible that exercise activity increased in the control group. However, consistent with participant self-report we saw evidence of fitness change only in the exercise group. Finally, it may be possible that the selected dose of exercise (duration and intensity) is insufficient or ill-suited to change amyloid accumulation. Future work should consider resistance training, or alternate intensities.

It is critical to note that the results of the study do not suggest that aerobic exercise is not beneficial. Aerobic exercise continues to have tremendous and unquestionable benefits for the body. Potential mechanisms for benefits observed with exercise include the upregulation of proteins involved in the clearance of amyloid [25,76] and reduction of systemic inflammation [77]. Tailoring exercise prescription may maximize the engagement of these processes. Our aerobic exercise group, for example, increased VO_2_ peak by 11%, with 11 individuals moving from a state of potentially impaired independence with a VO_2_ peak below 20 mL·kg^-1^·min^-1^ [78], to a more fully functional cardiorespiratory state of a VO_2_ peak above 20 mL·kg^-1^·min^-1^. Only one individual in the control group made that positive change, whereas 5 individuals in the group dropped below a VO_2_ peak of 20 mL·kg^-1^·min^-1^ during the study.

## Conclusions

The results of this trial do not support the hypothesis that 52 weeks of aerobic exercise influences amyloid burden in cognitively normal older adults. Additionally, secondary outcomes did not support prior work indicating that aerobic exercise benefits measures of brain health or cognition, at least in a cohort of cognitively normal older adults at elevated risk for Alzheimer’s due to elevated cerebral amyloid burden. The observed lack of cognitive or brain structure benefits, despite strong systemic cardiorespiratory effects of the intervention, suggests brain benefits of exercise reported in other studies are likely to be related to non-amyloid effects.

A large-scale, definitive trial is currently underway which will help to confirm or refute these findings [6].

## Supporting information

S1 Checklist CONSORT 2010 checklist of information to include when reporting a randomised trial*. (DOC)

S1 Table Primary and secondary outcomes of individuals with elevated amyloid. Mean and standard deviation. Mean and standard deviation. ^ Sample size for amyloid change Educ:25/Exercise:49. Sample size for change in VO_2_ peak Educ:24/Exercise:49. Sample size for change in brain volumes Educ:24/Education:47. Sample size for change in cognitive measures at baseline, week 26 and week 52 are Educ:26,26,25/Education:52,51,50. SUVR = Standard Uptake Value Ratio; VO_2_ peak = peak oxygen consumption during graded exercise test. * For amyloid, fitness and brain volume measures, a p-value from ordinary least squares regression adjusted for sex, age, and education comparing the change (baseline to week 52) between the two groups is given. For cognitive measures, a p-value for treatment by time interaction test from linear mixed models adjusted for sex, age, education, and amyloid status among per protocol subgroup is given.
(DOCX)

S2 Table Adverse events. (DOCX)

S1 File (DOCX)

## References

[1] Sabia S, Dugravot A, Dartigues JF, Abell J, Elbaz A, Kivimaki M, et al Physical activity, cognitive decline, and risk of dementia: 28 year follow-up of Whitehall II cohort study. BMJ. 2017;357:j2709 Epub 2017/06/24. 10.1136/bmj.j2709 28642251 PMC5480222

[2] Kramer AF, Hahn S, Cohen NJ, Banich MT, McAuley E, Harrison CR, et al Ageing, fitness and neurocognitive function. Nature. 1999;400(6743):418–9. 10.1038/22682 10440369

[3] Colcombe SJ, Kramer AF, Erickson KI, Scalf P, McAuley E, Cohen NJ, et al Cardiovascular fitness, cortical plasticity, and aging. Proc Nat Acad Sciences U S A. 2004;101(9):3316–21. 10.1073/pnas.0400266101 PMC373255 14978288

[4] Vidoni ED, Johnson DK, Morris JK, Van Sciver A, Greer CS, Billinger SA, et al Dose-response of aerobic exercise on cognition: a community-based, pilot randomized controlled trial. PLoS One. 2015;10(7):e0131647 10.1371/journal.pone.0131647 26158265 PMC4497726

[5] Maass A, Duzel S, Goerke M, Becke A, Sobieray U, Neumann K, et al Vascular hippocampal plasticity after aerobic exercise in older adults. Mol Psychiatry. 2015;20(5):585–93. Epub 2014/10/15. 10.1038/mp.2014.114 25311366

[6] Erickson KI, Grove GA, Burns JM, Hillman CH, Kramer AF, McAuley E, et al Investigating Gains in Neurocognition in an Intervention Trial of Exercise (IGNITE): Protocol. Contemp Clin Trials. 2019;85:105832 Epub 2019/08/30. 10.1016/j.cct.2019.105832 31465859 PMC6815730

[7] Norton S, Matthews FE, Barnes DE, Yaffe K, Brayne C. Potential for primary prevention of Alzheimer's disease: an analysis of population-based data. Lancet Neurol. 2014;13(8):788–94. 10.1016/S1474-4422(14)70136-X 25030513

[8] Rosenberg A, Ngandu T, Rusanen M, Antikainen R, Backman L, Havulinna S, et al Multidomain lifestyle intervention benefits a large elderly population at risk for cognitive decline and dementia regardless of baseline characteristics: The FINGER trial. Alzheimers Dement. 2018;14(3):263–70. Epub 2017/10/23. 10.1016/j.jalz.2017.09.006 29055814

[9] Burns JM, Cronk BB, Anderson HS, Donnelly JE, Thomas GP, Harsha A, et al Cardiorespiratory fitness and brain atrophy in early Alzheimer disease. Neurology. 2008;71(3):210–6. Epub 2008/07/16. 10.1212/01.wnl.0000317094.86209.cb 18625967 PMC2657657

[10] Honea RA, Thomas GP, Harsha A, Anderson HS, Donnelly JE, Brooks WM, et al Cardiorespiratory fitness and preserved medial temporal lobe volume in Alzheimer disease. Alzheimer Dis Assoc Disorder. 2009;23(3):188–97. Epub 2009/10/09. 10.1097/WAD.0b013e31819cb8a2 19812458 PMC2760037

[11] Erickson KI, Raji CA, Lopez OL, Becker JT, Rosano C, Newman AB, et al Physical activity predicts gray matter volume in late adulthood: The Cardiovascular Health Study. Neurology. 2010;75(16):1415–22. Epub 2010/10/15. 10.1212/WNL.0b013e3181f88359 [pii] 10.1212/WNL.0b013e3181f88359. 20944075 PMC3039208

[12] Weuve J, Kang JH, Manson JE, Breteler MM, Ware JH, Grodstein F. Physical activity, including walking, and cognitive function in older women. JAMA. 2004;292(12):1454–61. Epub 2004/09/24. 10.1001/jama.292.12.1454 15383516

[13] Krell-Roesch J, Feder NT, Roberts RO, Mielke MM, Christianson TJ, Knopman DS, et al Leisure-Time Physical Activity and the Risk of Incident Dementia: The Mayo Clinic Study of Aging. J Alzheimers Dis. 2018;63(1):149–55. Epub 2018/04/05. 10.3233/JAD-171141 29614667 PMC5900557

[14] Pereira AC, Huddleston DE, Brickman AM, Sosunov AA, Hen R, McKhann GM, et al An in vivo correlate of exercise-induced neurogenesis in the adult dentate gyrus. Proc Nat Acad Sciences U S A. 2007;104(13):5638–43. 10.1073/pnas.0611721104 17374720 PMC1838482

[15] Erickson KI, Voss MW, Prakash RS, Basak C, Szabo A, Chaddock L, et al Exercise training increases size of hippocampus and improves memory. Proc Nat Acad Sciences U S A. 2011;108(7):3017–22. Epub 2011/02/02. 1015950108 [pii] 10.1073/pnas.1015950108 21282661 PMC3041121

[16] Adlard PA, Perreau VM, Pop V, Cotman CW. Voluntary Exercise Decreases Amyloid Load in a Transgenic Model of Alzheimer's Disease. J Neurosci. 2005;25(17):4217–21. 10.1523/JNEUROSCI.0496-05.2005 15858047 PMC6725122

[17] Nation DA, Hong S, Jak AJ, Delano-Wood L, Mills PJ, Bondi MW, et al Stress, exercise, and Alzheimer's disease: a neurovascular pathway. Med Hypotheses. 2011;76(6):847–54. Epub 2011/03/15. S0306-9877(11)00085-5 [pii] 10.1016/j.mehy.2011.02.034 21398043 PMC3094492

[18] Radak Z, Ihasz F, Koltai E, Goto S, Taylor AW, Boldogh I. The redox-associated adaptive response of brain to physical exercise. Free Radical Res. 2014;48(1):84–92. 10.3109/10715762.2013.826352 23870001

[19] Radak Z, Hart N, Sarga L, Koltai E, Atalay M, Ohno H, et al Exercise plays a preventive role against Alzheimer's disease. 2010;J Alzheimers Dis. 20(3):777–83. Epub 2010/02/26. 10.3233/JAD-2010-091531 20182027

[20] Garcia-Mesa Y, Lopez-Ramos JC, Gimenez-Llort L, Revilla S, Guerra R, Gruart A, et al Physical exercise protects against Alzheimer's disease in 3xTg-AD mice. J Alzheimers Dis. 2011;24(3):421–54. Epub 2011/02/08. 847Q3J85285P1U07 [pii] 10.3233/JAD-2011-101635 21297257

[21] Leem YH, Lee YI, Son HJ, Lee SH. Chronic exercise ameliorates the neuroinflammation in mice carrying NSE/htau23. Biochem Biophys Res Commun. 2011;406(3):359–65. Epub 2011/02/19. S0006-291X(11)00238-5 [pii] 10.1016/j.bbrc.2011.02.046 21329662

[22] Maesako M, Uemura K, Kubota M, Kuzuya A, Sasaki K, Hayashida N, et al Exercise is more effective than diet control in preventing high fat diet-induced beta-amyloid deposition and memory deficit in amyloid precursor protein transgenic mice. J Biol Chem. 2012;287(27):23024–33. Epub 2012/05/09. 10.1074/jbc.M112.367011 22563077 PMC3391129

[23] Zhang J, Guo Y, Wang Y, Song L, Zhang R, Du Y. Long-term treadmill exercise attenuates Abeta burdens and astrocyte activation in APP/PS1 mouse model of Alzheimer's disease. Neurosci Lett. 2018;666:70–7. Epub 2017/12/17. 10.1016/j.neulet.2017.12.025 29246793

[24] Kang EB, Cho JY. Effects of treadmill exercise on brain insulin signaling and beta-amyloid in intracerebroventricular streptozotocin induced-memory impairment in rats. J Exerc Nutrition Biochem. 2014;18(1):89–96. Epub 2015/01/08. 10.5717/jenb.2014.18.1.89 25566443 PMC4241930

[25] Moore KM, Girens RE, Larson SK, Jones MR, Restivo JL, Holtzman DM, et al A spectrum of exercise training reduces soluble Abeta in a dose-dependent manner in a mouse model of Alzheimer's disease. Neurobiol Dis. 2016;85:218–24. 10.1016/j.nbd.2015.11.004 26563933

[26] Brown BM, Sohrabi HR, Taddei K, Gardener SL, Rainey-Smith SR, Peiffer JJ, et al Habitual exercise levels are associated with cerebral amyloid load in presymptomatic autosomal dominant Alzheimer's disease. Alzheimers Dement. 2017;13(11):1197–206. Epub 2017/05/16. 10.1016/j.jalz.2017.03.008 28501451 PMC5675772

[27] Liang KY, Mintun MA, Fagan AM, Goate AM, Bugg JM, Holtzman DM, et al Exercise and Alzheimer's disease biomarkers in cognitively normal older adults. Ann Neurol. 2010;68(3):311–8. 10.1002/ana.22096 20818789 PMC2936720

[28] Head D, Bugg JM, Goate AM, Fagan AM, Mintun MA, Benzinger T, et al Exercise Engagement as a Moderator of the Effects of APOE Genotype on Amyloid Deposition. Arch Neurol. 2012;69(5):636–43. Epub 2012/01/11. 10.1001/archneurol.2011.845 22232206 PMC3583203

[29] Brown BM, Peiffer JJ, Taddei K, Lui JK, Laws SM, Gupta VB, et al Physical activity and amyloid-beta plasma and brain levels: results from the Australian Imaging, Biomarkers and Lifestyle Study of Ageing. Mol Psychiatry. 2012;18(8):875–81. Epub 2012/08/15. 10.1038/mp.2012.107 22889922

[30] Daniele S, Pietrobono D, Fusi J, Iofrida C, Chico L, Petrozzi L, et al alpha-Synuclein Aggregates with beta-Amyloid or Tau in Human Red Blood Cells: Correlation with Antioxidant Capability and Physical Exercise in Human Healthy Subjects. Mol Neurobiol. 2018;55(3):2653–75. Epub 2017/04/20. 10.1007/s12035-017-0523-5 28421539

[31] Matthews DC, Davies M, Murray J, Williams S, Tsui WH, Li Y, et al Physical Activity, Mediterranean Diet and Biomarkers-Assessed Risk of Alzheimer's: A Multi-Modality Brain Imaging Study. Adv J Mol Imaging. 2014;4(4):43–57. Epub 2015/01/20. 10.4236/ami.2014.44006 25599008 PMC4294269

[32] Yoon DH, Lee JY, Shin SA, Kim YK, Song W. Physical Frailty and Amyloid-beta Deposits in the Brains of Older Adults with Cognitive Frailty. J Clin Med. 2018;7(7):169 Epub 2018/07/11. 10.3390/jcm7070169 29987248 PMC6068928

[33] Brown BM, Peiffer JJ, Martins RN. Multiple effects of physical activity on molecular and cognitive signs of brain aging: can exercise slow neurodegeneration and delay Alzheimer’s disease? Mol Psychiatry. 2013;18(8):864–74. 10.1038/mp.2012.162 23164816

[34] Law LL, Rol RN, Schultz SA, Dougherty RJ, Edwards DF, Koscik RL, et al Moderate intensity physical activity associates with CSF biomarkers in a cohort at risk for Alzheimer's disease. Alzheimers Dement (Amst). 2018;10:188–95. Epub 2018/03/13. 10.1016/j.dadm.2018.01.001 29527551 PMC5842318

[35] Okonkwo OC, Schultz SA, Oh JM, Larson J, Edwards D, Cook D, et al Physical activity attenuates age-related biomarker alterations in preclinical AD. Neurology. 2014;83(19):1753–60. Epub 2014/10/10. 10.1212/WNL.0000000000000964 25298312 PMC4239838

[36] Aisen PS, Andrieu S, Sampaio C, Carrillo M, Khachaturian ZS, Dubois B, et al Report of the task force on designing clinical trials in early (predementia) AD. Neurology. 2011;76(3):280–6. 10.1212/WNL.0b013e318207b1b9 21178097 PMC3034393

[37] Landau SM, Horng A, Jagust WJ. Memory decline accompanies subthreshold amyloid accumulation. Neurology. 2018;90(17):e1452–e1460. 10.1212/WNL.0000000000005354 29572282 PMC5921038

[38] McMillan CT, Chételat G. Amyloid “accumulators”. The next generation of candidates for amyloid-targeted clinical trials? Neurology. 2018;90(17):759–60. 10.1212/WNL.0000000000005362 29572279

[39] American College of Sports Medicine. ACSM's Guidelines for Exercise Testing and Prescription. 9th ed Baltimore, MD: Lippincott Williams & Wilkins; 2014.

[40] Vidoni ED, Bothwell RJ, Burns JM, Dwyer JR. Novel recruitment models will drive Alzheimer's trial success. Alzheimers Dement. 2018;14(1):117–9. 10.1016/j.jalz.2017.10.004 29156221 PMC5750126

[41] Mayer CJ, Steinman L, Williams B, Topolski TD, LoGerfo J. Developing a Telephone Assessment of Physical Activity (TAPA) questionnaire for older adults. Prev Chronic Dis. 2008;5(1):A24 Epub 2007/12/18 18082013 PMC2248772

[42] Burns JM, Johnson DK, Liebmann EP, Bothwell RJ, Morris JK, Vidoni ED. Safety of disclosing amyloid status in cognitively normal older adults. Alzheimers Dement. 2017;13(9):1024–30. Epub 2017/03/07. 10.1016/j.jalz.2017.01.022 28263740 PMC5582024

[43] Morris JC. The Clinical Dementia Rating (CDR): current version and scoring rules. Neurology. 1993;43(11):2412–4. 10.1212/wnl.43.11.2412-a 8232972

[44] Weintraub S, Besser L, Dodge HH, Teylan M, Ferris S, Goldstein FC, et al Version 3 of the Alzheimer Disease Centers' Neuropsychological Test Battery in the Uniform Data Set (UDS). Alzheimer Dis Assoc Disord. 2018;32(1):10–7. 10.1097/WAD.0000000000000223 29240561 PMC5821520

[45] Harn NR, Hunt SL, Hill J, Vidoni E, Perry M, Burns JM. Augmenting Amyloid PET Interpretations With Quantitative Information Improves Consistency of Early Amyloid Detection. Clin Nucl Med. 2017;42(8):577–81. Epub 2017/06/03. 10.1097/RLU.0000000000001693 28574875 PMC5491352

[46] Clark CM, Pontecorvo MJ, Beach TG, Bedell BJ, Coleman RE, Doraiswamy PM, et al Cerebral PET with florbetapir compared with neuropathology at autopsy for detection of neuritic amyloid-beta plaques: a prospective cohort study. Lancet Neurol. 2012;11(8):669–78. 10.1016/S1474-4422(12)70142-4 22749065

[47] Joshi AD, Pontecorvo MJ, Clark CM, Carpenter AP, Jennings DL, Sadowsky CH, et al Performance characteristics of amyloid PET with florbetapir F 18 in patients with alzheimer's disease and cognitively normal subjects. J Nuc Med. 2012;53(3):378–84. Epub 2012/02/15. 10.2967/jnumed.111.090340 22331215

[48] Vidoni ED, Van Sciver A, Johnson DK, He J, Honea R, Haines B, et al A community-based approach to trials of aerobic exercise in aging and Alzheimer's disease. Contemp Clin Trials. 2012;33(6):1105–16. Epub 2012/08/21. 10.1016/j.cct.2012.08.002 22903151 PMC3468654

[49] Morris JK, Vidoni ED, Johnson DK, Van Sciver A, Mahnken JD, Honea RA, et al Aerobic exercise for Alzheimer's disease: A randomized controlled pilot trial. PLoS One. 2017;12(2):e0170547 10.1371/journal.pone.0170547 28187125 PMC5302785

[50] Dustman RE, Ruhling RO, Russell EM, Shearer DE, Bonekat HW, Shigeoka JW, et al Aerobic Exercise Training and Improved Neuropsychological Function of Older Individuals. Neurobiol Aging. 1984;5(1):35–42. 10.1016/0197-4580(84)90083-6 6738784

[51] Reuter M, Schmansky NJ, Rosas HD, Fischl B. Within-subject template estimation for unbiased longitudinal image analysis. Neuroimage. 2012;61(4):1402–18. Epub 2012/03/21. 10.1016/j.neuroimage.2012.02.084 22430496 PMC3389460

[52] Goodglass H, Kaplan E. The assessment of aphasia and related disorders. Philadelphia: Lea & Febiger; 1983.

[53] Armitage SG. An analysis of certain psychological tests used in the evaluation of brain injury. Psychological Monographs. 1946;60:1–48.

[54] Wechsler D. Wechsler Memory Scale III: Administration and scoring manual. Orlando, FL: Psychological Corporation; 1997.

[55] Stroop J. Studies of interference in serial verbal reactions. J Exp Psychol. 1935;18:643–62.

[56] Grober E, Buschke H, Crystal H, Bang S, Dresner R. Screening for dementia by memory testing. Neurology. 1988;38:900–3. 10.1212/wnl.38.6.900 3368071

[57] Ekstrom R, French J, Harman H, Dermen D. Kit of Factor-Referenced Cognitive Tests. Princeton NJ: Electronic Testing Service; 1976 Available from: https://www.ets.org/Media/Research/pdf/Kit_of_Factor-Referenced_Cognitive_Tests.pdf.

[58] Rinne JO, Brooks DJ, Rossor MN, Fox NC, Bullock R, Klunk WE, et al 11C-PiB PET assessment of change in fibrillar amyloid-beta load in patients with Alzheimer's disease treated with bapineuzumab: a phase 2, double-blind, placebo-controlled, ascending-dose study. Lancet Neurol. 2010;9(4):363–72. Epub 2010/03/02. 10.1016/S1474-4422(10)70043-0 20189881

[59] Stewart AL, Verboncoeur CJ, McLellan BY, Gillis DE, Rush S, Mills KM, et al Physical Activity Outcomes of CHAMPS II: A Physical Activity Promotion Program for Older Adults. J Gerontol A: Biol Sci Med Sci. 2001;56(8):M465–70. 10.1093/gerona/56.8.m465 11487597 PMC1780022

[60] Fjell AM, Walhovd KB, Fennema-Notestine C, McEvoy LK, Hagler DJ, Holland D, et al One-year brain atrophy evident in healthy aging. J Neurosci. 2009;29(48):15223–31. Epub 2009/12/04. 10.1523/JNEUROSCI.3252-09.2009 19955375 PMC2827793

[61] Guadagni V, Drogos L, Tyndall A, Davenport M, Anderson T, Eskes G, et al Aerobic exercise improves cognition and cerebrovascular regulation in older adults Neurology. 2020;94(21):e2245–e3357 Epub 05/13/2020. 10.1212/WNL.0000000000009478 32404355 PMC7357295

[62] Baker LD, Frank LL, Foster-Schubert K, Green PS, Wilkinson CW, McTiernan A, et al Aerobic exercise improves cognition for older adults with glucose intolerance, a risk factor for Alzheimer's disease. J Alzheimers Dis. 2010;22(2):569–79. Epub 2010/09/18. 10.3233/JAD-2010-100768 20847403 PMC3049111

[63] Baker LD, Frank LL, Foster-Schubert K, Green PS, Wilkinson CW, McTiernan A, et al Effects of aerobic exercise on mild cognitive impairment: a controlled trial. Arch Neurol. 2010;67(1):71–9. Epub 2010/01/13. 67/1/71 [pii] 10.1001/archneurol.2009.307 20065132 PMC3056436

[64] Frederiksen KS, Madsen K, Andersen BB, Beyer N, Garde E, Hogh P, et al Moderate- to high-intensity exercise does not modify cortical beta-amyloid in Alzheimer's disease. Alzheimers Dement (N Y). 2019;5:208–15. Epub 2019/06/15. 10.1016/j.trci.2019.04.006 31198839 PMC6556817

[65] Petersen RC, Wiste HJ, Weigand SD, Rocca WA, Roberts RO, Mielke MM, et al Association of Elevated Amyloid Levels With Cognition and Biomarkers in Cognitively Normal People From the Community. JAMA Neurol. 2016;73(1):85–92. Epub 2015/11/26. 10.1001/jamaneurol.2015.3098 26595683 PMC4710552

[66] Schwarz CG, Gunter JL, Lowe VJ, Weigand S, Vemuri P, Senjem ML, et al A Comparison of Partial Volume Correction Techniques for Measuring Change in Serial Amyloid PET SUVR. J Alzheimers Dis. 2019;67(1):181–95. Epub 2018/11/27. 10.3233/JAD-180749 30475770 PMC6398556

[67] Sevigny J, Chiao P, Bussiere T, Weinreb PH, Williams L, Maier M, et al The antibody aducanumab reduces Abeta plaques in Alzheimer's disease. Nature. 2016;537(7618):50–6. Epub 2016/09/02. 10.1038/nature19323 27582220

[68] Machulda MM, Pankratz VS, Christianson TJ, Ivnik RJ, Mielke MM, Roberts RO, et al Practice effects and longitudinal cognitive change in normal aging vs. incident mild cognitive impairment and dementia in the Mayo Clinic Study of Aging. Clin Neuropsychol. 2013;27(8):1247–64. 10.1080/13854046.2013.836567 24041121 PMC3869900

[69] Machulda MM, Hagen CE, Wiste HJ, Mielke MM, Knopman DS, Roberts RO, et al Practice effects and longitudinal cognitive change in clinically normal older adults differ by Alzheimer imaging biomarker status. Clin Neuropsychol. 2017;31(1):99–117. Epub 2016/10/12. 10.1080/13854046.2016.1241303 27724156 PMC5408356

[70] Liu-Ambrose T, Nagamatsu LS, Graf P, Beattie BL, Ashe MC, Handy TC. Resistance training and executive functions: a 12-month randomized controlled trial. Arch Intern Med. 2010;170(2):170–8. Epub 2010/01/27. 170/2/170 [pii] 10.1001/archinternmed.2009.494 20101012 PMC3448565

[71] Billinger SA, Vidoni ED, Morris JK, Thyfault JP, Burns JM. Exercise Test Performance Reveals Support of the Cardiorespiratory Fitness Hypothesis. J Aging Phys Act. 2016;25(2):240–246. 10.1123/japa.2015-0321 27705069 PMC5374040

[72] Vidoni ED, Szabo-Reed A, Kang C, Shaw AR, Perales-Puchalt J, Grove G, et al The IGNITE trial: Participant recruitment lessons prior to SARS-CoV-2. Contemp Clin Trials Commun. 2020;20:100666 Epub 2020/10/15. 10.1016/j.conctc.2020.100666 33052319 PMC7544598

[73] Blocker EM, Fry AC, Luebbers PE, Burns JM, Perales-Puchalt J, Hansen DM, et al Promoting Alzheimer's Risk-Reduction through Community-Based Lifestyle Education and Exercise in Rural America: A Pilot Intervention. Kans J Med. 2020;13:179–85. 32695261 PMC7363174

[74] Perales-Puchalt J, Shaw A, McGee JL, Moore WT, Hinton L, Resendez J, et al Preliminary Efficacy of a Recruitment Educational Strategy on Alzheimer's Disease Knowledge, Research Participation Attitudes, and Enrollment Among Hispanics. Hisp Health Care Int. 2020;18(3):144–9. Epub 2019/12/17. 10.1177/1540415319893238 31840539 PMC7293919

[75] Shaw AR, Perales-Puchalt J, Bright B, Moore WT, Robinson M, Hill CV, et al Recruitment of Older African Amricans in Alzheimers Disease Clinical Trials Using a Community Based Research Approach. medRxiv. 2020 10.1101/2020.07.16.20155556. PMC9514712 36281671

[76] Di Loreto S, Falone S, D'Alessandro A, Santini S Jr., Sebastiani P, Cacchio M, et al Regular and moderate exercise initiated in middle age prevents age-related amyloidogenesis and preserves synaptic and neuroprotective signaling in mouse brain cortex. Exp Gerontol. 2014;57:57–65. Epub 2014/05/20. 10.1016/j.exger.2014.05.006 24835196

[77] Nichol KE, Poon WW, Parachikova AI, Cribbs DH, Glabe CG, Cotman CW. Exercise alters the immune profile in Tg2576 Alzheimer mice toward a response coincident with improved cognitive performance and decreased amyloid. J Neuroinflammation. 2008;5:13 Epub 2008/04/11. 10.1186/1742-2094-5-13 18400101 PMC2329612

[78] Cress ME, Meyer M. Maximal voluntary and functional performance levels needed for independence in adults aged 65 to 97 years. Phys Ther. 2003;83(1):37–48. Epub 2002/12/24. 12495411

