# Supplementary material for: Effect of aerobic exercise on amyloid accumulation in preclinical Alzheimer’s: A 1-year randomized controlled trial
Source: PLoS One. 2021 Jan 14;16(1):e0244893. doi: 10.1371/journal.pone.0244893 (PMC7808620; doi:10.1371/journal.pone.0244893)
Supplement: S1 Table — Mean and standard deviation. Mean and standard deviation. ^ Sample size for amyloid change Educ:25/Exercise:49. Sample size for change in VO2 peak Educ:24/Exercise:49. Sample size for change in brain volumes Educ:24/Education:47. Sample size for change in cognitive measures at baseline, week 26 and week 52 are Educ:26,26,25/Education:52,51,50. SUVR = Standard Uptake Value Ratio; VO2 peak = peak oxygen consumption during graded exercise test. * For amyloid, fitness and brain volume measures, a p-value from ordinary least squares regression adjusted for sex, age, and education comparing the change (baseline to week 52) between the two groups is given. For cognitive measures, a p-value for treatment by time interaction test from linear mixed models adjusted for sex, age, education, and amyloid status among per protocol subgroup is given. (DOCX) [file pone.0244893.s002.docx]

| S1 Table. Outcomes of individuals with elevated amyloid | | | | | | | |
| --- | --- | --- | --- | --- | --- | --- | --- |
|  |  | Intent-to-Treat (n-79^) | | | Per-protocol (n=65^) | | |
|  | Timepoint | Standard of Care Education  Control (n=27) | Aerobic Exercise (n=52) | p-value* | Standard of Care Education  Control (n=27) | Aerobic Exercise (n=16) | p-value* |
| Global Amyloid (SUVR) | Baseline | 1.27 (0.17) | 1.31 (0.16) | 0.52 | 1.26 (0.16) | 1.31 (0.17) | 0.81 |
|  | Week 52 | 1.27 (0.16) | 1.32 (0.15) |  | 1.27 (0.16) | 1.3 (0.15) |  |
|  | Change | 0.01 (0.04) | 0.02 (0.07) |  | 0.01 (0.04) | -0.02 (0.07) |  |
| VO_2_ peak (mL·kg^-1^·min^-1^) | Baseline | 23.2 (5.1) | 21.8 (5.1) | <0.01 | 23.5 (4.8) | 22.4 (4.9) | <0.01 |
|  | Week 52 | 24.1 (4.9) | 23.7 (5.9) |  | 24.1 (4.9) | 24.7 (5.7) |  |
|  | Change | 0.2 (2.3) | 1.8 (2.2) |  | 0.20 (2.3) | 2.4 (2.1) |  |
| Whole Brain (mL) | Baseline | 1055.15 (127.60) | 1049.85 (97.19) | 0.26 | 1055.15 (127.65) | 1047.97 (83.35) | 0.22 |
|  | Week 52 | 1052.72 (128.36) | 1045.52 (96.30) |  | 1052.72 (128.4) | 1043.30 (85.12) |  |
|  | Change | -2.4 (6.80) | -4.33 (7.83) |  | -0.24 (6.8) | -4.68 (7.10) |  |
| Hippocampal Volume (mL) | Baseline | 7.52 (1.02) | 7.42 (0.77) | 0.22 | 7.52 (1.02) | 7.47(0.69) | 0.20 |
|  | Week 52 | 7.43 (1.05) | 7.37 (0.77) |  | 7.43 (1.05) | 7.43 (0.67) |  |
|  | Change | -0.91 (0.16) | -0.05 (0.089) |  | -0.091 (0.16) | -0.05 (0.09) |  |
| Executive Function Composite | Baseline | -0.03 (0.46) | -0.029 (0.46) | 0.89 | -0.001 (0.33) | -0.01 (0.40) | 0.91 |
|  | Wk 26 | -0.06 (0.64) | -0.06 (0.635) |  | -0.05 (0.36) | -0.02 (0.62) |  |
|  | Wk 52 | -0.07 (0.64) | -0.07 (0.64) |  | -0.03 (0.43) | -0.01 (0.63) |  |
| Verbal Memory Composite | Baseline | -0.06 (0.92) | -0.06 (0.92) | 0.55 | 0.02 (0.91) | -0.03 (0.88) | 0.44 |
|  | Wk 26 | -0.25 (1.02) | -0.25 (1.02) |  | -0.04 (1.04) | -0.26 (0.93) |  |
|  | Wk 52 | -0.20 (0.97) | -0.20 (0.97) |  | -0.03 (1.07) | -0.23 (0.83) |  |
| Visuospatial Composite | Baseline | -0.08 (0.63) | -0.08 (0.63) | 0.75 | -0.08 (0.58) | -0.07 (0.60) | 0.59 |
|  | Wk 26 | -0.13 (0.71) | -0.13 (0.71) |  | -0.02 (0.64) | -0.07 (0.69) |  |
|  | Wk 52 | -0.13 (0.65) | -0.13 (0.65) |  | -0.08 (0.59) | -0.11 (0.64) |  |

Mean and standard deviation.

^ Sample size for amyloid change Educ:25/Exercise:49. Sample size for change in VO2 Educ:24/Exercise:49. Sample size for change in brain volumes Educ:24/Education:47. Sample size for change in cognitive measures at baseline, week 26 and week 52 are Educ:26,26,25/Education:52,51,50.

* For amyloid, fitness and brain volume measures, a p-value from ordinary least squares regression adjusted for sex, age, and education comparing the change (baseline to week 52) between the two groups is given. For cognitive measures, a p-value for treatment by time interaction test from linear mixed models adjusted for sex, age, education, and amyloid status among per protocol subgroup is given.
