# Supplementary material for: Effect of aerobic exercise on amyloid accumulation in preclinical Alzheimer’s: A 1-year randomized controlled trial
Source: PLoS One. 2021 Jan 14;16(1):e0244893. doi: 10.1371/journal.pone.0244893 (PMC7808620; doi:10.1371/journal.pone.0244893)
Supplement: S2 Table — (DOCX) [file pone.0244893.s003.docx]

| S2 Table. Adverse events | | | |
| --- | --- | --- | --- |
|  | Timepoint | Education Control | Aerobic Exercise |
| Related to Intervention | Mild | NA | 31 |
|  | Moderate | NA | 2 |
|  | Severe | NA | 0 |
| Unrelated to Intervention | Mild | 10 | 48 |
|  | Moderate | 3 | 12 |
|  | Severe | 5 | 7 |
