# Supplementary material for: Effect of aerobic exercise on amyloid accumulation in preclinical Alzheimer’s: A 1-year randomized controlled trial
Source: PLoS One. 2021 Jan 14;16(1):e0244893. doi: 10.1371/journal.pone.0244893 (PMC7808620; doi:10.1371/journal.pone.0244893)
Supplement: S1 File — (DOCX) [file pone.0244893.s004.docx]

**Effect of Aerobic Exercise on Pathophysiology of PreClinical Alzheimer’s Disease**

**Principal Investigator: Jeffrey Burns, MD**

**Amendment 4**

**Version 5.0**

**Date 22 April 2016**

**[Note: This document contains the Research Protocol, Plan, and Human Subjects Protection information submitted as part of the overall RO1 grant application initially submitted to the NIH and reviewed by the HSC in August of 2012.]**

**SPECIFIC AIMS**

Our long-term research goal is to develop and test strategies to prevent Alzheimer’s disease (AD). There is an increasing interest in the role of exercise in the prevention and treatment of AD given the growth of the elderly population and subsequent increase in age-related disorders. Until disease-modifying AD therapies are available, risk factor reduction will remain the cornerstone of dementia prevention.^[1](#_ENREF_1)^ Accumulating evidence suggests that aerobic exercise may protect against cognitive decline and dementia.^[2-14](#_ENREF_2)^ Additionally, animal studies suggest exercise may modify AD neuropathological burden.^[15-19](#_ENREF_15)^ Our NIH-funded work has demonstrated that aerobic fitness is positively associated with brain volume and cognitive performance in early AD.^[20](#_ENREF_20), [21](#_ENREF_21)^ Moreover, we have extensive experience conducting rigorous NIH-funded clinical trials to test the effects of aerobic exercise on the brain (R01AG034614, R01AG033673).

The advent of amyloid imaging creates an opportunity for identifying individuals in the presumptive presymptomatic phase of the disease, when interventions are likely to have the greatest impact.^[22](#_ENREF_22)^ Approximately 30% of cognitively normal older adults have asymptomatic cerebral amyloidosis and thus meet the NIA and Alzheimer’s Association research criteria for “preclinical AD.” The concept of preclinical AD posits that cerebral amyloid deposition in cognitively normal adults represents a presymptomatic stage of AD and individuals with preclinical AD currently represent the earliest feasible stage for trials of AD prevention.

This study examines the effects of aerobic exercise on AD pathophysiology (amyloid burden) and associated “downstream” neurodegeneration (regional atrophy) and cognitive decline in preclinical AD. The proposal extends our ongoing work on the role of exercise to promote a healthier brain and capitalizes on our new status as an AD Center (KU ADC; P30AG035982). We will examine the effects of a 52-week aerobic exercise program in 120 individuals, 70 with preclinical AD and 50 with nonelevated amyloid. We will use existing clinical research infrastructure of the KU ADC to recruit 400 sedentary, cognitively normal (CDR 0) individuals age 65 and older for screening with Florbetapir PET imaging to enroll 70 preclinical AD participants (defined as cognitively normal participants with elevated cerebral amyloid) and 50 individuals with non-elevated cerebral amyloid. Participants will be randomized in a 2:1 ratio to either aerobic exercise or control. Exercise training will occur in a community setting through the network of KUMC approved facilities.

**Aim 1: Determine the effect of aerobic exercise on amyloid burden in individuals with preclinical AD (cognitively normal with elevated cerebral amyloid) and those without elevated amyloid.** We hypothesize that 52 weeks of aerobic exercise will be associated with lower amyloid burden as measured by Florbetapir PET imaging. Our primary amyloid burden measure will be mean change from baseline to 52 weeks in Florbetapir cortical-to-cerebellar ratio averaged across 6 regions of interest (frontal, temporal, parietal, anterior cingulate, posterior cingulate, and precuneus).

**Aim 2:** **Determine if aerobic exercise attenuates structural brain changes in preclinical AD and those without elevated amyloid.** We hypothesize that aerobic exercise will attenuate hippocampal atrophy (1° imaging outcome measure), an area of accelerated atrophy in preclinical AD. Additionally, we will examine the effect of exercise on secondary measures of precuneal and whole-brain atrophy.

**Aim 3: Determine the effects of aerobic exercise on cognition in individuals with preclinical AD and those without elevated amyloid**. We hypothesize that aerobic exercise will provide cognitive benefits in our primary outcome measure of executive function and in secondary measures of memory and visuospatial function. Additionally, we expect that improvements in memory will correlate with structural preservation in hippocampus.

**Exploratory Aim: Examine potential underlying mechanisms relating exercise with brain health.** Our findings suggest that AD-related cognitive impairment and brain atrophy are associated with systemic changes, including accelerated sarcopenia and bone loss,^[23-26](#_ENREF_23)^ which are responsive to physical activity interventions. Thus, we will explore exercise-related changes in serum markers that may mediate these systemic manifestations including BDNF, anabolic hormones (insulin, insulin-like growth factor) and inflammatory markers (c-reactive protein, interleukin-6, TNF-alpha). We will explore exercise-related change in these markers and their relationship with changes in AD biomarkers and body composition.

The unprecedented growth of the elderly population^[27](#_ENREF_27)^ and increasing prevalence of AD^[28](#_ENREF_28)^ underscores the need for addressing AD prevention strategies.^[29](#_ENREF_29)^ Delaying the onset of AD by 5 years could cut AD incidence by 50% and reduce the US health care burden by $50 billion annually. This proposal will provide important preliminary data assessing potential disease-modifying benefits of exercise on AD pathophysiology in the putative earliest stages of disease and inform the design of more definitive studies of exercise as an AD prevention strategy. Our experience in community-based exercise trials, new status as an ADC, and partnership with Avid Radiopharmaceuticals provides a unique opportunity to efficiently achieve these aims.

**RESEARCH STRATEGY**

**Significance / Background**

Preclinical AD: A New Target for AD Prevention Strategies

In 2011, the NIA and Alzheimer's Association defined research criteria to provide a common rubric to advance the study of the preclinical stages of AD and stimulate studies in early intervention.^[30](#_ENREF_30)^ These criteria define preclinical AD as the presence of cerebral amyloid (measured by amyloid imaging or low CSF Aß_42_) in individuals without cognitive or clinical evidence of dementia. The concept of preclinical AD postulates AD neuropathology accumulates in the brain for years before the emergence of cognitive deficits and that progressive deterioration will culminate in dementia if the individual continues to live. Studies supporting the concept of preclinical AD demonstrate cerebral amyloid in nondemented individuals is associated with AD-like endophenotypes of progressive cognitive decline, brain atrophy, and altered brain function (reviewed below). Thus, preclinical AD represents an important therapeutic target for AD prevention strategies.

AD Pathophysiological Cascade

AD is defined neuropathologically as the presence of cerebral beta-amyloid (Aβ) deposits and neurofibrillary tangles. Biomarker studies suggest AD pathophysiologic changes likely begin years prior to the emergence of clinical symptoms and follow a temporal ordering of events beginning with cerebral amyloid deposition, followed by elevations in CSF tau, and later the development of “downstream” AD-related neurodegeneration (reduced temporo-parietal metabolism and brain atrophy). Amyloid deposition alone may not be sufficient to produce AD clinical symptoms and may require the presence of concomitant tau-based pathology or neurodegenerative processes (i.e., atrophy) to produce clinical symptoms.^[31](#_ENREF_31)^ Convergent evidence from AD biomarker studies suggests amyloid deposition may be a necessary and early component of the AD pathophysiological process.^[32](#_ENREF_32)^ Further research is necessary to define the precise role of amyloid in the pathoetiology of AD, although amyloid imaging allows the identification of key early AD pathophysiological events in individuals who remain without functional or cognitive impairments.

Amyloid Imaging

The molecular imaging of amyloid deposits has promise as a potential biomarker for AD and may allow the identification of individuals in the preclinical stages of the illness. Pittsburgh compound-B (PIB) is the most widely studied radioligand although its use is restricted by its short half-life. Other molecular imaging agents have been developed using longer-lived F18 radiolabels (such as FDDNP, Florbetapir, and Florbetaben) making this technology more widely available. The current proposal will use Florbetapir PET imaging, which has been validated at autopsy as reflecting the presence and quantity of amyloid pathology.^[33](#_ENREF_33)^ We have extensive experience with the use of Florbetapir PET through national multi-site trials (Bapineuzumab, ADNI, IGIV). Additionally, we have a close working relationship with Avid Pharmaceuticals (see letter of support) and Avid will provide the costs of 200 PET scans and 485 Florbetapir doses.

Amyloid Imaging as Biomarker for Preclinical AD

The clinical significance of cerebral amyloid plaques remains imprecisely defined although early studies suggest asymptomatic cerebral amyloid is not benign. Autopsy,^[34-36](#_ENREF_34)^ CSF,^[37](#_ENREF_37)^ and amyloid imaging studies^[38-41](#_ENREF_38)^ suggest cerebral amyloid is present in 20 – 40% of cognitively normal older adults at levels similar to those seen in AD. The presence of cerebral amyloid is predictive of AD risk in individuals with MCI^[42-45](#_ENREF_42)^ and higher mean cortical binding values of PIB predicted progression to AD in a cohort of 159 cognitively normal older adults.^[46](#_ENREF_46)^ Although studies of the relationship of cerebral amyloid and cognition in nondemented individuals have been mixed,^[44](#_ENREF_44)^ multiple studies have reported that elevated cerebral amyloid is associated with modest differences in cognitive performance^[47](#_ENREF_47), [48](#_ENREF_48)^ and more robust longitudinal decline in cognition^[39](#_ENREF_39), [49](#_ENREF_49), [50](#_ENREF_50)^ and function.^[51](#_ENREF_51)^

Additionally, neuroimaging suggest cerebral amyloid is associated with structural and functional brain changes in areas implicated early in AD. Analyses from the ADNI dataset suggest that lower CSF amyloid^[52](#_ENREF_52)^ and increased PIB retention^[44](#_ENREF_44)^ are associated with greater whole brain atrophy and regional atrophy in the medial temporal lobe and precuneus.^[31](#_ENREF_31), [44](#_ENREF_44), [52](#_ENREF_52)^ Studies in other cohorts have observed elevated PIB retention is associated cross-sectionally with lower brain volume^[39](#_ENREF_39), [53](#_ENREF_53)^ and with longitudinal rates of global brain atrophy.^[54](#_ENREF_54), [55](#_ENREF_55)^ Additionally, increased cerebral amyloid in nondemented older adults is associated with atrophy in specific regions involved early in the AD process,^[48](#_ENREF_48), [56-60](#_ENREF_56)^ most commonly the hippocampus^[48](#_ENREF_48), [59](#_ENREF_59)^ and the posterior cingulate / precuneus.^[56](#_ENREF_56), [57](#_ENREF_57), [60](#_ENREF_60)^ Asymptomatic amyloid elevated individuals have also demonstrated evidence of disrupted functional connectivity.^[61-63](#_ENREF_61)^ Thus, substantial data suggests preclinical AD is associated with cognitive decline, brain atrophy, and altered brain function, supporting the concept of preclinical AD as a novel target population for testing AD prevention strategies.

**Rationale for Exercise as an AD Prevention Strategy**

Exercise and physical activity have a biologically plausible and temporal relationship with coronary heart disease,^[64](#_ENREF_64)^ atherosclerosis,^[65](#_ENREF_65)^ stroke,^[66](#_ENREF_66)^ type 2 diabetes, some cancers, and all-cause mortality.^[67](#_ENREF_67)^ As described below, a wealth of data indicates exercise also positively impacts brain health.

*Exercise and Cognition:* Cross-sectional studies suggest physical activity is positively associated with cognition, particularly in executive and visuospatial function.^[68-70](#_ENREF_68)^ Additionally, epidemiologic studies suggest that exercise may prevent cognitive decline and dementia. Physical activity in midlife is associated with a reduced risk of developing AD in late-life.^[71](#_ENREF_71)^ Multiple longitudinal studies report a relationship between self-reported exercise and cognitive decline.^[5-7](#_ENREF_5), [13](#_ENREF_13), [72](#_ENREF_72), [73](#_ENREF_73)^ We found greater baseline levels of aerobic fitness are associated with slower dementia progression in AD (n=37) and a trend to less cognitive decline in nondemented older adults (n=53).^[74](#_ENREF_74)^

*Neuroimaging Evidence:* Evidence from MRI studies suggests that exercise, and associated aerobic fitness levels, may attenuate age- and AD-related brain changes. Higher levels of aerobic fitness are associated cross-sectionally with mitigation of age-related declines in brain density.^[75](#_ENREF_75)^ In randomized controlled trials, aerobic exercise promotes brain plasticity^[76](#_ENREF_76)^ and attenuates hippocampal atrophy while improving spatial memory.^[77](#_ENREF_77)^ Our studies suggest aerobic fitness in individuals in the earliest stages of AD (n=57) is associated with whole brain volume (r=0.54, p<0.001),^[78](#_ENREF_78)^ most strongly in the hippocampus and parietal regions, areas implicated in early AD.^[79](#_ENREF_79)^ Our longitudinal analyses suggest that decline in aerobic fitness is associated with greater atrophy in the parahippocampus in early AD (n=37) and greater frontal and basal ganglia atrophy in nondemented controls (n=53).^[74](#_ENREF_74)^ These results suggest physical exercise may have disease-modifying effects on AD pathophysiology.

Additionally, we have performed studies demonstrating associations of body composition (BMI, lean mass, bone density) with AD biomarkers, brain atrophy and cognition, providing additional support for exercise in delaying the functional onset of AD. AD is associated with weight loss in the preclinical stages of the disease.^[80](#_ENREF_80)^ Using the ADNI cohort, we found that lower BMI was associated with the presence and burden of cerebral amyloid and CSF tau in nondemented and MCI participants.^[81](#_ENREF_81)^ Using more sensitive measures (DEXA), we found that AD is predominantly associated with loss of lean mass^[23](#_ENREF_23)^ and bone density,^[25](#_ENREF_25), [26](#_ENREF_26)^ which in turn are related to measures of AD progression (cognitive performance and brain atrophy). Physical activity has systemic effects on anabolic signaling and inflammation, which are shared pathophysiologic factors associated with AD, sarcopenia, and osteoporosis. Although the causal relationship of these variables remains unclear, this data supports the hypothesis that systemic effects of exercise may modify the co-varying AD-related declines in body composition and measures of neurodegeneration.

Potential Mechanisms

There are a number of potential mechanisms that may relate exercise with brain health. Increased physical activity may have a trophic effect on the brain, particularly the hippocampus. For instance, exercise is associated with increased brain-derived neurotrophic factor (BDNF)^[82](#_ENREF_82)^ and other important neurochemicals^[83](#_ENREF_83)^ supporting brain growth and survival. Exercise stimulates neurogenesis,^[84](#_ENREF_84)^ enhances resistance to brain insults, ^[85-87](#_ENREF_85)^ increases synaptic plasticity,^[88](#_ENREF_88)^ and promotes brain vascularization.^[89](#_ENREF_89), [90](#_ENREF_90)^ Additionally, exercise reduces vascular risk factors (heart disease,^[64](#_ENREF_64)^ atherosclerosis,^[65](#_ENREF_65)^ stroke,^[66](#_ENREF_66)^ diabetes^[91-96](#_ENREF_91)^) that place an individual at risk for dementia, vascular dementia, and AD.

*Exercise and Amyloid:* In addition to the wealth of data on the positive effects of exercise on the brain, animal studies indicate exercise may reduce amyloid burden and modify AD pathophysiology through direct effects on amyloid precursor protein metabolism^[15-17](#_ENREF_15)^ and indirect effects on neurotrophic factors, neuroinflammation, and oxidative stress. Multiple transgenic mouse models of AD have shown that physical activity in mice reduces neuropathological burden,^[15](#_ENREF_15)^ increases neuronal plasticity,^[82](#_ENREF_82)^ improves spatial learning and memory, ^[18](#_ENREF_18), [82](#_ENREF_82)^, and modulates inflammatory processes. ^[19](#_ENREF_19), [83](#_ENREF_83)^. Taken together, animal studies suggest that voluntary exercise may reduce the production of beta-amyloid and modify the AD pathophysiological cascade.

Studies in humans assessing the effect of physical activity on AD pathophysiology are sparse, although exercise in cognitively normal older adults is associated with lower cerebral amyloid deposition (as assessed by both PIB imaging and CSF AB).^[97](#_ENREF_97)^ Additionally, exercise may interact with ApoE4 to influence amyloid deposition whereby increased physical activity is associated with lower amyloid deposition in E4 carriers.^[98](#_ENREF_98)^ These findings are consistent with longitudinal studies reporting physical activity may have greater benefits on cognition in ApoE4 carriers,^[99-104](#_ENREF_99)^ although similar studies have been mixed.^[105-108](#_ENREF_105)^ Thus, limited human data suggests physical activity is associated with lower cerebral amyloid deposition although these findings need confirmation in rigorous controlled trials.

**KU ADC Exercise Trial Experience**

We have extensive experience with performing rigorous exercise trials in older adults with and without dementia, including two ongoing randomized controlled trials to be completed in early 2013. As of February 2012, we have enrolled a total of 100 individuals into these 6-month intervention trials including individuals with AD (n=28) and cognitively normal older adults (n=72).

The Trial of Exercise on Aging and Memory (TEAM) study (R01AG034614) is a 6-month dose-response study assessing the cognitive benefits of 50%, 100%, and 150% of the recommended exercise dose (150min / week) in nondemented older adults. As of February 2011, we have enrolled 72 participants (goal 100) and study enrollment will be completed in mid 2012. Adherence to the weekly exercise duration goal has been high (89%) and retention excellent (51 of 53 [96%] completed the 6-month intervention). Of the 51 individuals who have completed the intervention, aerobic fitness as measured by peak oxygen consumption (VO_2_peak) increased following a dose-response function (F=11.5,p<.001): VO_2_peak decreased in the no exercise group (-1.1ml/kg/min, -5.0%) and increased in the 50% group (1.6ml/kg/min, +6.8%), 100% group (1.5ml/kg/min, +7.3%) and the 150% group (+2.48ml/kg/min, +13.0%). Preliminary cognitive analyses indicate benefits in the executive domain (composite score of Stroop Test, Digit Symbol, Trails B, and Category Fluency; F=4.08, p<.05) in a dose response pattern. Standardized scores in the no exercise group were unchanged (-.08), improved slightly in the 50% group (+.17), and were not statistically different in the 100% and 150% groups (.27 and .25). Our preliminary results suggest that physiologic adaptations to exercise increase in a dose response pattern while the cognitive benefits appear to plateau at the 100% dosage. Thus, 150 minutes a week optimally balances physiologic effects, cognitive benefits and participant burden.

We have also adapted our exercise protocols for AD. In 2007 we piloted a 4-month aerobic exercise program in 6 individuals with AD using a community exercise facility. Exercise was tolerated well in this group of older adults with AD. In 2010, we began the Alzheimer’s Disease Exercise Program Trial (ADEPT), a 6-month randomized trial of aerobic exercise vs. stretching control assessing cognitive and structural MRI outcomes (R01AG033673). Currently, we have enrolled 28 participants and anticipate reaching 60 participants by January 2013. Ten participants have completed the 6-month intervention with one drop out.

Exercise trials in older adults are complex, burdensome (to participants and the study team), and require a well-coordinated multi-disciplinary approach. Our record for recruiting, characterizing, and exercising older adults is excellent. Overall adherence to 6 months of exercise or a rigorous control intervention has been high: of 100 participants (n=72 nondemented and 29 with AD) enrolled into aerobic exercise interventions, 61 have completed the intervention, 36 are actively exercising, and 3 have dropped out (5% of completers). This history combined with our extensive experience performing longitudinal imaging studies^[74](#_ENREF_74),[109](#_ENREF_109),[110](#_ENREF_110)^ uniquely positions us to rigorously measure the biological impact of aerobic exercise on AD pathophysiology in preclinical AD.

**INNOVATION**

In 2010, an NIH State-of-the-Science Conference examined data on AD prevention^[111](#_ENREF_111)^ and concluded the current data is insufficient to draw firm conclusions on the association of modifiable risk factors, including physical exercise, with cognitive decline and AD. The greatest limitation of the data is the largely observational nature of the studies, limiting the ability to discern association from causation. No randomized controlled trials (RCT) were identified by the panel evaluating the effect of physical activity on delaying AD onset. The panel reinforced the critical need for RCTs to investigate strategies to maintain cognitive function in at-risk adults.

The advent of amyloid imaging creates an opportunity for identifying individuals in the presumptive presymptomatic phase of the disease, when interventions are likely to have the greatest impact.^[22](#_ENREF_22)^ Moreover, amyloid imaging provides an opportunity to examine how exercise impacts the pathological hallmark of AD, cerebral amyloid deposition. Additionally, our use of structural neuroimaging biomarkers will provide additional proof-of-concept data regarding potential disease modifying benefits of aerobic exercise (i.e., atrophy). While larger clinical trials with standard clinical trial endpoints (i.e., dementia / AD onset) will be essential for definitive proof of the role of exercise in AD prevention, this study will provide important data on the biological effects of aerobic exercise in high-risk individuals and provide a foundation for more definitive multi-site trials.

Until disease-modifying therapeutics are available, risk reduction strategies will remain the cornerstone of AD prevention strategies. Aerobic exercise offers a low-cost, low-risk intervention that is widely-available and may have disease modifying effects. There is a need for rigorous data demonstrating and defining the efficacy of exercise in slowing age-related cognitive decline and enhancing functional independence. Demonstrating aerobic exercise alters AD pathophysiology in preclinical AD would have enormous public health implications. The study’s implications may impact public health policy by encouraging the public to adapt more active lifestyles and stimulate the development of effective exercise delivery programs to enhance initiation and maintenance of physical activity interventions in at-risk older adults.

**APPROACH**

The current proposal extends our ongoing efforts of the KU Alzheimer’s Disease Center (KU ADC) to determine the role of aerobic exercise in brain health. This project also advances the KU ADC’s larger mission of understanding the role of metabolism in aging and AD. Dr. Burns initiated the AD program in 2004 and has been supported by NIH funding to assess the role of fitness in brain aging and AD progression (R03AG026374, K23NS058252, R21AG029615) and two ongoing clinical trials assessing how aerobic exercise influences cognition in healthy aging (R01AG034614) and the progression of AD (R01AG033673).

**Sample and Recruitment History:** The KU ADC has established a history of excellent recruitment for investigator-initiated studies and ADCS-sponsored clinical trials drawing on our extensive experience with outreach efforts, recruitment databases and advertising. The greater Kansas City area (Missouri and Kansas) is home to 2.3 million people with 2004 estimates indicating over 11% of the population is 65 years and older. Since 2004, the KU AD clinical research program has recruited and characterized over 500 participants using the standard UDS Registry assessment for a variety of investigator-initiated studies. Within five months of being funded as an ADC in August 2011, we have enrolled and characterized 108 participants into our longitudinal ADC cohort. We will extend our well-developed recruitment and clinical infrastructure for screening 400 nondemented older adults over 3 years to meet our enrollment goal of 70 individuals with preclinical AD and 50 with nonelevated amyloid.

**Study Procedures**

This is a randomized controlled trial of 52 weeks of aerobic exercise on sedentary individuals with preclinical AD (n=70) and those with non-elevated cerebral amyloid (n=50) The KU ADC will screen a total of 400 cognitively normal older adults with Florbetapir PET to achieve our enrollment goal. Participants will be randomized in a 2:1 ratio to active aerobic exercise vs. control using randomization sequences generated by the study statistician (Mahnken). Exercise interventions will be rigorously controlled and supervised in a community-based network of KUMC approved facilities across the Kansas City area. Given our history of 5% drop out over a 6-month exercise trial, we are projecting a 15% annual dropout rate. Participants will have Florbetapir PET and MRI before and after 52 weeks of exercise. Cognitive testing will be completed at baseline, 26-, and 52 weeks. Figure 1 provides an overview of the screening and study events described in more detail below.

**Timeline and Overview of Study Events**

***Timeline:*** We anticipate initiating screening of participants within 2 months of funding and beginning enrollment and the intervention within 4 months. Enrollment will be ongoing with screening of 400 participants occurring over three years (~ 3 participants a week) with enrollment completed in the middle of the 4^th^ year of the grant. The final participants will complete the 52-week intervention in the middle of the 5^th^ year, allowing time to complete data analyses and prepare manuscripts.

***Screening:*** The KU ADC will screen a total of 400 participants 65 years of age and older to establish a sufficient cohort (n~120 given an estimated 30% will have evidence of cerebral amyloid) to enroll 70 preclinical AD and 50 amyloid nonelevated participants into the intervention. Screening will include a standard clinical and cognitive evaluation (KU ADC Registry or similar cognitive impairment screening) and Florbetapir PET.

***Baseline Evaluation***: The baseline evaluation will consist of the following outcome assessments (methodological details provided below) : brain MRI, cognitive testing, physical function assessment (aerobic fitness testing for VO_2_ peak, DEXA scan for body composition, and physical function), , physical activity, and phlebotomy.

***26-Week Evaluation:*** At 26 weeks (+/- 1 week),, participants will return for an in-person evaluation to repeat cognitive testing and assessments of physical activity.

***52-Week Evaluation:*** At 52 weeks (+/- 1 week), participants will return to complete Florbetapir PET, brain MRI, cognitive testing, physical function assessment (VO_2_ peak, DEXA, physical function), physical activity assessment, and phlebotomy.

***Telephone Checks:*** Participants will be contacted by phone for formal review of medication changes, medical history, and adverse events in the first week of initiating the exercise intervention and again at approximately weeks 6, 12, 18, 32, 39, and 46 during the intervention. These formal assessments will complement the routine assessments performed by exercise instructors that briefly assess new medical or exercise-related adverse events. Additionally, the telephone checks will encourage communication between the study team and the participants and encourage compliance with the intervention.

***Blinding:*** Raters (psychometrician, exercise physiologist) who are blinded to the participant’s intervention group (exercise vs. control) will perform outcome assessments. Dr. Burns will be unblinded and perform safety assessments and address safety concerns or adverse events.

See the Study Timeline below for procedures and timing of visits.

**
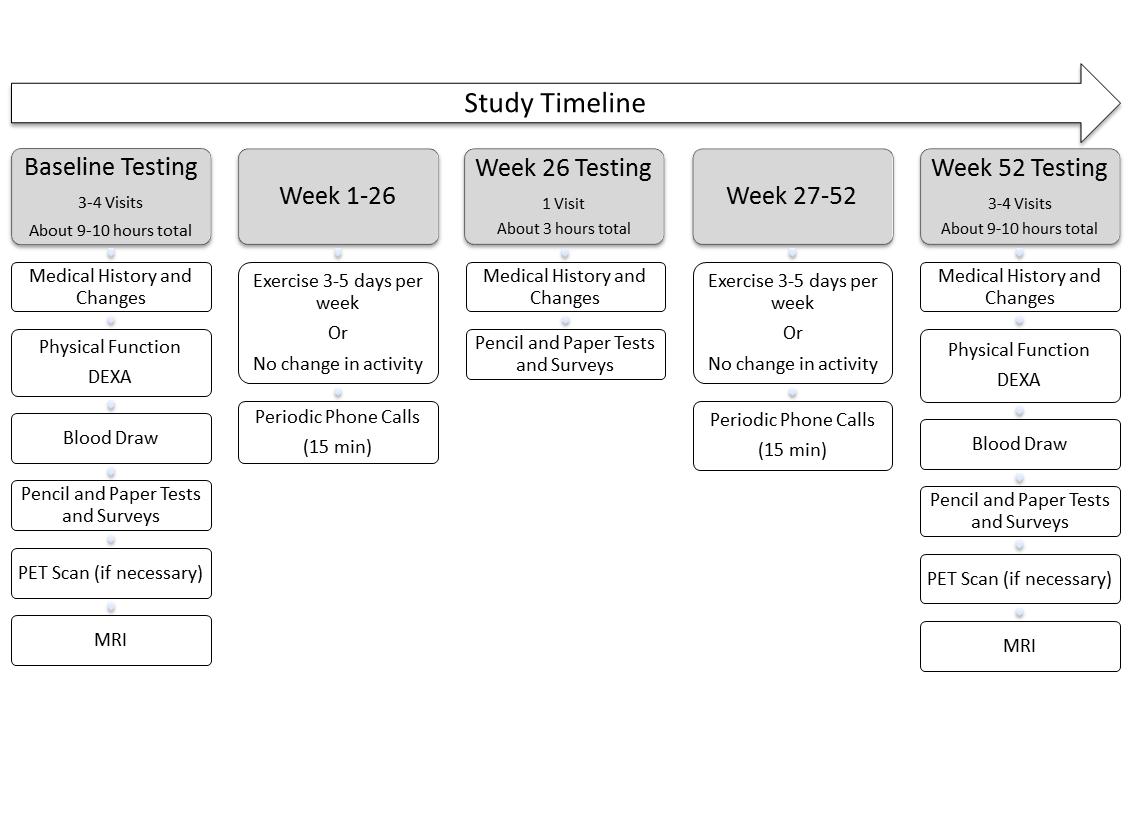
**

**Detailed Methodology**

**Screening Procedures**

The KU ADC infrastructure will support screening using standard IRB-approved procedures currently in use. The KU ADC was funded in August 2011 and is establishing a longitudinal cohort of 200 nondemented and 200 MCI / AD participants. By February 2012, the KU ADC enrolled a total of 108 participants (53 nondemented, 21 MCI, 31 AD, 3 non-AD participants). As the KU ADC is charged with maintaining a cohort of 200 nondemented participants, funding for this protocol will augment the ADC infrastructure to support recruitment and characterization (clinical, cognitive, Florbetapir PET, described below) of an additional 200 participants to meet the screening goal of 400 nondemented participants.

We expect the need to screen 400 participants to identify approximately 120 individuals (30% of the screening population) who meet the following inclusion / exclusion criteria. Consenting to participate in the exercise trial will occur after the screening process, although participants will be well-informed of the primary purpose of the screening procedures. Although we do not expect all potential participants (n=120) to agree to participate, we expect that a majority (100 of the 120; 83%) will participate in the 52 week exercise trial.

Inclusion criteria include

- CDR 0 (nondemented)
- Age 65 or older
- Florbetapir PET evidence of cerebral amyloidosis
- Non-elevated Florbetapir PET scan algorithm computerized measuring a mean quantitative standard uptake value ratio equal to or greater than 1.0 for 6 regions of interest.
- Sedentary or underactive by the Telephone Assessment of Physical Activity
- Stable doses of medications for 30 days.
- Clinician judgment regarding subject’s health status and likelihood to successfully complete the 1-year exercise intervention

Exclusion criteria include

- Clinically significant major psychiatric disorder (e.g., Major Depressive Disorder) according to DSM-IV criteria or significant psychiatric symptoms that could impair the completion of the study
- Clinically-significant systemic illness that may affect safety or completion of the study
- History of clinically-evident stroke
- Clinically-significant infection within the last 30 days
- Active cardiac condition (e.g. angina, myocardial infarction, atrial fibrillation) or pulmonary condition in the past 2 years that, in the investigator’s opinion, could pose a safety risk to the participant—unless cleared for exercise by the participant’s primary care physician or cardiologist.
- Uncontrolled hypertension within the last 6 months
- History of cancer in the last 5 years (except non-metastatic basal or squamous cell carcinoma)
- History of drug or alcohol abuse as defined by DSM-IV criteria within the last 2 years
- Insulin-dependent diabetes mellitus
- Significant pain or musculoskeletal disorder prohibiting participation in an exercise program
- Unwillingness to undergo or contraindication to brain MRI scan.
- History within the last 5 years of primary or recurrent malignant disease with the exception of resected localized cutaneous squamous cell carcinoma, basal cell carcinoma, cervical carcinoma, or prostate cancer.

***Non-elevated Amyloid Group:*** We will recruit individuals with non-elevated amyloid PET scans who have a mean SUVR (in 6 regions of interest) of 1.0 and greater to be enrolled into the trial as part of the non-elevated amyloid group. Based on our first 84 non-elevated participants screened for the study, this represents about 1/3^rd^ of those with non-elevated amyloid PET scans. Participants will be recruited by letter or phone calls to those who have been previously screened and meet the criteria or during in-person screening visits until a total of 50 nonelevated participants are enrolled.

SUVR (Standard Uptake Value Ratio) represents a ratio of the retention of radiotracer in the brain compared to the patient’s control region of the cerebellum. A higher is believed to reflect higher levels of amyloid in the brain although in this group of individuals who do not evidence of amyloid deposition given their non-elevated status, the relevance of the SUVR value remains uncertain. Nevertheless, this group is of scientific interest given some early data suggesting the group may be enriched with individuals who accumulate amyloid over time.

Rationale for not disclosing SUVR values: Participants will be informed that they are in a group representing 1/3^rd^ of those with non-elevated scans that have been selected as eligible to participate based on computer algorithm. Our current practice is not to communicate information on patient’s SUVR values and we believe this continues to be important until more data is available to suggest that SUVR values are shown to be clinically relevant. There is currently no consensus or evidence that within non-elevated participants SUVR values have clinical relevance. It remains unclear that an SUVR value, especially in the non-elevated range, provides any predictive information on risk. Additionally, it remains unclear whether the sensitivity and precision of the measure influences how these values can be interpreted. Not disclosing SUVR values to participants is the current practice for the national Alzheimer’s Disease Neuroimaging Initiative. We have a scientific interest in the group of individuals in the top third of SUVR measures as there is early evidence that those with higher amyloid burdens may accumulate amyloid at a more rapid rate than others. Whether individuals who accumulate amyloid are at higher risk of cognitive change is not yet known although given that our primary aim is to assess the impact of aerobic exercise on amyloid accumulation.

Given the burden and expense of amyloid scanning, MRI scanning, and a one-year exercise program, it is not feasible to enroll a sample of all non-elevated participants.

*Clinical and Cognitive Evaluation:* All participants will have completed a standard cognitive screening by a trained rater. A neuropsychological test battery is performed by a psychometrician. Clinical and cognitive data are reviewed at a consensus diagnostic conference attended by ADC clinicians, neuropsychologist, and nurses. Participants without evidence of functional or cognitive decline who are sedentary (Telephone Assessment of Physical Activity^[114](#_ENREF_114)^) will next be evaluated with a Florbetapir PET scan. If more than 90 days have elapsed between enrollment and PET scanning, the participant will have to be rescanned. Eligibility is determined based on the initial screening PET scan (not a repeat amyloid PET).

*Florbetapir PET:* Florbetapir PET scans will be obtained and visually read by a trained radiologist and two trained clinicians using quantitative analyses performed as an adjunct to the visual read. This technique combines a visual and quantitative assessment into one read referred to as VisQ (i.e., Visual and Quantitative reading). The final determination of amyloid elevated vs. non-elevatedis based on the majority of the three independent VisQ results.

Given estimates of cerebral amyloid deposition in approximately 30% of nondemented older adults, we expect to identify approximately 120 of the 400 screened participants will have elevated amyloid. Of these with elevated amyloid, we anticipate 85% will be interested in participating in the aerobic exercise protocol. We expect 70% to be nonelevated (~280 of the total 400 planned to be screened) and of these 1/3^rd^ will be eligible for enrollment in the nonelevated group. We will enroll 50 of these participants into the nonelevated group.

**Rationale for Exercise Dose and Duration:** Aerobic exercise is a mainstay of all public health recommendations. Walking at moderate intensity is the most common form of exercise for older adults and is easily adopted and widely prescribed. Thus it is highly relevant to public health recommendations. The current exercise protocol is built on the success of our ongoing Trial of Exercise for Aging and Memory (R01AG034614). 150 minutes a week represents the recommended weekly duration by the American College of Sports Medicine and therefore has important “real-world” implications.^[115](#_ENREF_115)^ Further, based on our preliminary results, this dose achieved our best balance between fitness, cognitive benefits, and participant adherence

The study duration of 52 weeks was chosen for three reasons: 1) feasibility, 2) to maximize physiologic benefits of exercise, and 3) evidence from natural history studies suggesting individuals with preclinical AD have measurable changes in brain atrophy and cognition over 52 weeks. First, our experience suggests 12 months is achievable based on high retention and adherence rates to our 6-month intervention. Second, physiologic adaptations to exercise (VO2, muscle mass) occur at 3-4 months consistently^[116](#_ENREF_116)^ but it remains unclear how long is needed for brain benefits. A meta-analysis of exercise ^[3](#_ENREF_3)^ suggests durations greater than 6 months are associated with more cognitive benefits and 52 weeks of exercise is associated with structural and functional brain change.^[77](#_ENREF_77), [117](#_ENREF_117)^ Third, early preclinical AD studies have observed decline in cognition and brain structure in 52 week longitudinal studies.^[77](#_ENREF_77)^ These expected changes in controls will enhance the study’s power to observe exercise-related effects on AD pathophysiology and cognition. Thus, a 52 week intervention is feasible and will enhance the likelihood of observing exercise-related biological changes in the brain.

**Aerobic Exercise Intervention**

Upon successful completion of baseline evaluations, participants randomized to the aerobic intervention group will exercise 150 minutes over 3 to 4 days per week for 52 weeks with supervision in a local KUMC approved facility. Through our current trials, we have a strong relationship with the 14 greater Kansas City YMCAs (see letter of support) and several additional facilities with which we have a memorandum of understanding concerns conducting research. Each center is staffed by trained exercise personnel with experience in the older adult population and personal training certification through the National Commission for Certifying Agencies.

Aerobic exercise will consist primarily of walking on a treadmill, although participants can choose to exercise on a separate aerobic modality to reduce repetitive stress and boredom. Participants will gradually increase their aerobic exercise duration from 60 minutes in the first week to 150 minutes by the sixth week (increasing 21 minutes in duration a week). This progressive duration increase allows previously sedentary participants to become accustomed to increased activity and minimizes injury. All sessions include 5-minute warm-up and cool-down periods. Each subject will wear a heart rate monitoring chest strap during each exercise session. Beginning at 40 – 55% of Heart Rate Reserve (% of difference between maximal and resting), target heart rate zones will be increased by 10% of HRR every 3 months. Subjects unable to exercise continuously on the treadmill will perform intermittent training until the target duration is reached. Aerobic fitness training logs will be maintained for duration of exercise,.. Adherence to the weekly duration goals will be followed and recorded by assessing the participant’s attendance and ability to meet weekly exercise duration goals.

Exercise Supervision: We will build on the strengths of current protocols from both the TEAM and ADEPT studies. Direct supervision by exercise instructors will occur for all exercise sessions until the weekly duration target of 150 minutes a week is reached (not before 6 weeks). We introduce more flexibility in scheduling exercise after this if the participant is consistently and safely meeting their target duration goals by allowing unsupervised exercise sessions at the approved facility at times when exercise trainers may not be available (i.e. early morning, nights, and weekends). Participants are still required to have at least one directly supervised exercise session per week to maintain contact with program staff and encourage adherence to the program. Unsupervised exercise sessions are conducted similarly to supervised exercise and session data will be reviewed by the exercise instructor at the weekly supervised session.

Adherence and Retention: We use community based exercise facilities given our experience that distance to the training site makes recruitment for exercise programs difficult; thus, utilizing community-based facilities increases our base of recruitable subjects by providing easier access and shorter commutes. Additionally, the national network is potentially an ideal platform for a future multi-site trial. Each participant receives a one-year membership to an approved facility. Exercise protocol adherence is directly monitored as the percentage of the weekly exercise duration attained. If participation falls below the target, we work to encourage participants and identify problems that may be barriers to exercise compliance. Our history suggests high adherence to exercise goals (89%) and low dropout rates over 6-months (5%). We recognize and anticipate that participants may need to miss several exercise sessions due to travel plans, family obligations, etc. Heart rate monitors are issued to participants to record exercise data if traveling or for exercise on their own. Participants are strongly discouraged from making these accommodations more than 4 weeks total over the course of the study. We also encourage retention and adherence through other activities and incentives (small gifts).

Training Plan for Exercise Instructors: Members of our exercise intervention team have extensive experience performing exercise interventions Prior to beginning the trial and periodically thereafter, the exercise intervention team holds informational training meetings with exercise trainers to provide an overview to the study and discuss the training protocols. During these training sessions, the exercise intervention team meets with the exercise instructors to ensure adequate training, compliance with the training plan, and quality data collection. Additionally, the exercise intervention team is available by phone to answer questions or address unforeseen problems. Turnover of exercise instructors is historically very low although new trainers complete the training protocol prior to involvement in the intervention.

Quality Assurance for Training and Data Collection: While a site is active, a member of the exercise intervention team makes site visits regularly to collect data forms, review quality of data collection (e.g., completeness and accuracy of data entry) and ensure competency in the standard application of the exercise protocol through observations of the exercise instructors. If procedural discrepancies are identified, these are then addressed with the instructors.

Safety: One trainer supervises a maximum of four participants at one time. If the participant is deemed a fall risk, the trainers are prepared to provide 1:1 supervisor to participant supervision. All of the treadmills are equipped with handrails and emergency stop switches. All of the trainers are Adult CPR certified and trained in the use of portable defibrillators which are available at all of the facilities.

**Control Group**

*Standard-of-care education (SOC)* – All study participants randomized to the control group will receive education from study staff about the importance of maintaining a healthy and active lifestyle. Each enrollee will receive our SOC education based on a standardized script along with standardized literature, “Exercise and Physical Activity: Your Everyday Guide from the National Institutes on Aging” and the "KUADC Promoting Healthy Brain Aging.” These booklets provide vetted and reliable information for older adults on how to exercise.  Participants may ask questions of study staff at that time. Participants randomized to the control group will receive no further counseling on physical activity.


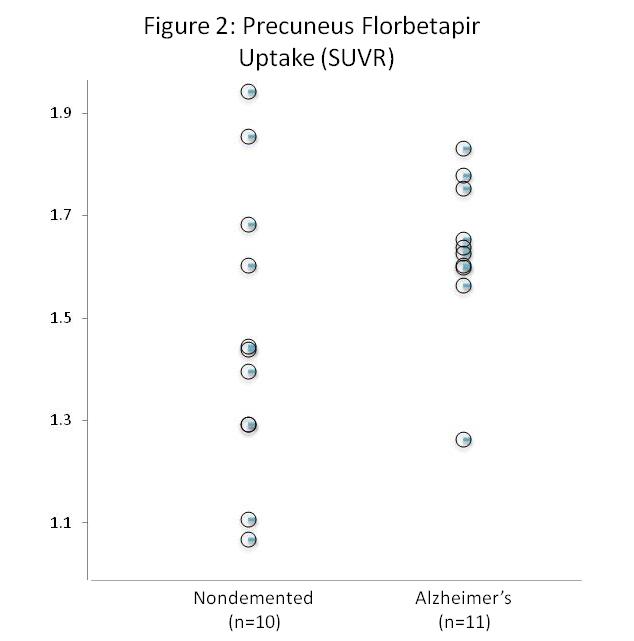


**Aim 1: Determine the effect of aerobic exercise on amyloid burden in individuals with preclinical AD (cognitively normal with elevated cerebral amyloid) and those without elevated amyloid.**

Rationale: Amyloid imaging has promise as a potential biomarker for identifying the preclinical stages of AD and is being used as an outcome measure to assess biological effects of therapies in clinical trials. This study uses amyloid imaging to 1) identify individuals with early AD pathophysiologic changes (preclinical AD) who are likely at higher risk of developing AD, and 2) to assess potential disease-modifying effects of exercise on early AD pathophysiology. Amyloid imaging techniques have demonstrated sensitivity to change in response to therapeutic agents (Bapineuzumab, see power analyses below).^[119](#_ENREF_119)^ Additionally, amyloid burden increases longitudinally in nondemented older adults, with the greatest increase observed in individuals who are amyloid elevated at baseline (i.e., similar to our preclinical AD cohort).^[120](#_ENREF_120)^ As animal studies^[15-17](#_ENREF_15)^ and indirect human data^[97](#_ENREF_97), [98](#_ENREF_98)^ suggests exercise modifies the AD pathophysiologic cascade, we hypothesize that aerobic exercise will be associated with reduced cortical amyloid burden over 52 weeks compared to controls.

Preliminary Experience: We have performed 21 Florbetapir PET scans through several clinical trials (ADNI, IGIV, Bapineuzumab). Our nondemented sample (n=10, mean age 82) had 4 of 10 individuals with an amyloid burden similar to that seen in our AD sample (n=11, mean age 76), 10 of 11 whom demonstrated elevated amyloid burden (Figure 2).

Data Acquisition: Florbetapir PET imaging will be obtained at screening and after 52 weeks of exercise (+/- 1 week). Subjects will have a catheter placed for IV administration of Florbetapir F 18 Injection. Vital signs will be taken in a supine position immediately prior to administration of Florbetapir (within 5 minutes). Subjects will receive a single IV bolus of Florbetapir (370 MBq) followed by brain PET imaging of 10 minutes duration, approximately 50 minutes post-dose injection. Adverse events will be continuously monitored during the imaging session and a participant will have follow-up phone call within 2 business days after the imaging session to confirm patient well-being and to collect information about any new adverse events.

Outcomes: Images will be evaluated qualitatively and quantitatively. Data will be submitted to Avid for qualitative analysis. Images will be visually examined by a trained radiologist or nuclear medicine physician who is blinded to the subject diagnosis and will be reported as either Aβ elevated (AD-like) or Aβ non-elevated (not AD-like). Qualitative interpretation will be performed in the following manner: the images will be reviewed using a black-white scale with maximum intensity of the scale set to the maximum intensity of all brain pixels. A elevated scan will have at least one area where the grey matter is more intense than adjacent white matter or at least 2 brain areas where the grey white contrast is reduced or absent. For quantitative evaluation, standard uptake values (SUV) will be calculated using SPM software in 6 cortical areas (frontal cortex, temporal cortex, parietal cortex, precuneus, anterior and posterior cingulate) and the cerebellum. SUV ratios (SUVR) for cortical target areas relative to the cerebellum will also be calculated and a global mean SUVR will be calculated from the average across all cortical target areas.

Statistical Analyses for Aim 1: This is a two-arm, 2:1 randomized, controlled clinical trial comparing 52 weeks of aerobic exercise to control intervention. Based on drop out rates from those who have completed our exercise trials (2 of 53 [4%] dropped out of the TEAM study and 1 of 11 [9%] dropped out of ADEPT), we conservatively estimate that 15% of the subjects will drop out of the study, leaving approximately 85 subjects for analysis. We will perform analyses based on intent-to-treat to maintain the benefits of the randomization. Per protocol analyses will also be conducted among the subjects that complete a minimum of 80% of their sessions. To further assess the response to aerobic exercise, we will conduct analyses that replace the primary explanatory factor (treatment group) with continuous measures of increase (change from baseline) in aerobic fitness (V0_2_peak). This will allow us to assess the aerobic fitness hypothesis which states that aerobic fitness gains mediate neural benefits of exercise.

We hypothesize that 52 weeks of aerobic exercise will lower amyloid burden as measured by Florbetapir PET imaging. Amyloid burden will be measured at baseline and at 52 weeks as a ratio of standard uptake values in six regions of interest to the cerebellum. Our primary amyloid burden outcome measure will be the 52-week change in Florbetapir cortical-to-cerebellar ratio averaged across these six regions of interest. The primary intent-to-treat analysis for amyloid burden will compare the change from baseline to 52 weeks between our two treatment groups using the two-sample t-test. Similar analyses will be conducted for each of the ratios for the six regions of interest individually (secondary measures), and for per-protocol analyses described above. Given the single primary measure for this aim, no multiplicity adjustment will be made and secondary endpoints will be clearly indicated as such in resulting manuscripts. To evaluate the appropriateness of our approach (two-sample t-test), residual analysis will be conducted. This will include visual inspection of the residuals and comparison of the estimated standard deviation across groups to assess homogeneity of variances. If these diagnostics indicate concerns with our parametric approach, appropriate alternative strategies, such as nonparametric methods like the Wilcoxon rank-sum test or data transformation, will be utilized. Ordinary least squares (OLS) regression models will be used for the per protocol analyses that will assess increase in aerobic fitness as continuous measures with amyloid burden outcomes. Model assessment for these OLS models will also be conducted by assessing model assumptions through residual analyses (e.g., predicted versus residual plots, quantile-quantile [q-q] plots, etc.).

**Aim 2:** **Determine if aerobic exercise attenuates structural brain changes in preclinical AD and those without elevated amyloid.**

Rationale: Magnetic resonance (MR) based volumetric studies are increasingly used as surrogate outcome measures of disease^[121](#_ENREF_121)^ to more efficiently demonstrate an intervention’s disease-modifying effect. Our primary focus will be on the effect of exercise in modifying regional atrophy in the hippocampus with secondary outcome measures including rates of whole-brain and precuneal atrophy. The hippocampus is an important structure in mediating memory processes^[122](#_ENREF_122)^ and is among the earliest affected regions in AD.^[123](#_ENREF_123)^ Additionally, atrophy of the hippocampus has been observed in preclinical AD and is associated with asymptomatic cerebral amyloid deposition.^[48](#_ENREF_48), [59](#_ENREF_59)^ We are also interested in the effect of exercise on regional atrophy in the precuneus, an important node in the default mode network that, along with the hippocampus, appears to be vulnerable to structural and functional decline associated with asymptomatic cerebral amyloidosis.^[61](#_ENREF_61), [124-128](#_ENREF_124)^ Importantly, these structures may be responsive to exercise: a randomized controlled trial of 52 weeks of exercise attenuated hippocampal atrophy^[77](#_ENREF_77)^ while physical activity levels are associated with hippocampal and precuneus volumes in nondemented older adults.^[129](#_ENREF_129)^

Preliminary Experience: Our studies suggest that aerobic fitness (VO_2_peak) in the earliest stages of AD is associated with whole brain atrophy^[78](#_ENREF_78)^ and atrophy in the hippocampus^[79](#_ENREF_79)^ while decline in aerobic fitness over 2 years is associated with greater atrophy in the parahippocampus.^[74](#_ENREF_74)^ Although our imaging data is limited to aerobic fitness measures (rather than an exercise intervention), the data suggest that exercise to maintain or enhance aerobic fitness may have disease modifying effects on AD-related structural changes. Additionally, our imaging methods are sensitive to hippocampal and precuneus atrophy as we observed annualized rates of atrophy in the hippocampus (1.72% [2.7]) and precuneus (0.56% [0.40]) in nondemented older adults (n=63) over two years. We expect to see greater rates of atrophy in our preclinical AD participants which will enhance our power to observe exercise-related effects.

Data Acquisition: We employ the core T1-weighted MPRAGE and FLAIR sequences used by ADNI2 with a Siemens 3T Skyra MRI scanner at KUMC’s Hoglund Brain Imaging Center. Our primary imaging measures will come from high-resolution structural images (MP-RAGE; 1x1x1.2mm voxels; TR=2300ms, TE=2.98ms, TI=1900, FOV=256, Flip angle=9°). We will also acquire additional secondary imaging outcome assessments using arterial spin labeling (ASL), diffusion tensor imaging and resting-state functional connectivity data using standard ADNI protocols. These secondary measures will allow us to explore exercise effects on regional blood flow (ASL), structural white matter changes (DTI), and functional connectivity (BOLD) given our prior experience with these measures.^[74](#_ENREF_74), [130](#_ENREF_130)^

Outcomes: Hippocampal and precuneus volumes at baseline and 52 weeks will be assessed by processing images via the Freesurfer pipeline^[131](#_ENREF_131)^ optimized for longitudinal analysis. Longitudinal processing uses a subject-specific template for segmentation^[132](#_ENREF_132)^ to initialize the longitudinal image processing to increase repeatability and statistical power. Automated Talairach transformation and segmentation of the cortex, subcortical white matter and deep gray matter volumetric structures (including hippocampus) is performed and change scores can be calculated to assess atrophy.

In addition to our primary imaging volumetric outcome measures of precuneus and hippocampal atrophy, we will assess measures of whole brain, gray matter, and white matter atrophy from the Freesurfer analysis. Additionally, we will use voxel based morphometry (VBM8 for SPM) to explore exercise-related effects on regional brain atrophy. VBM examines the whole brain and selected regions of interest in an unbiased way with high sensitivity to identify small changes in brain structure over time. We have extensive experience with VBM methods.^[21](#_ENREF_21), [74](#_ENREF_74), [109](#_ENREF_109), [133](#_ENREF_133), [134](#_ENREF_134)^ This approach has been used effectively in exercise studies of similar duration in nondemented older adults^[118](#_ENREF_118)^ and will allow us to test our hypotheses while generating important pilot data for refining plans for a more definitive study of exercise in AD.

Statistical Analyses for Aim 2: We hypothesize that aerobic exercise will attenuate atrophy in the precuneus and hippocampus, two regions consistently identified with atrophy in preclinical AD and the earliest stages of AD. Volume measures of the hippocampus, precuneus, and of the whole brain (continuous variables) will be collected at baseline and 52 weeks. The primary measure of brain atrophy will be hippocampal volume.

The primary intent-to-treat analysis for atrophy will compare the change from baseline to 52 weeks between our two treatment groups. The two-sample t-test of the observed changes will be used for this comparison. Similar models will be generated for precuneus volume and whole brain volume, and for the per-protocol analyses described above. Given a single primary measure for this aim, no multiplicity adjustment will be made and secondary endpoints will be clearly indicated as such in resulting manuscripts. Model assessment and alternative approaches will follow as described in Aim 1.

**Aim 3: Determine the effects of aerobic exercise on cognition in individuals with preclinical AD and those without elevated amyloid.**.

Rationale: Substantial evidence, including our own (see background above), exists demonstrating that aerobic exercise has a preferential effect on cognition, particularly in executive functioning.^[69](#_ENREF_69), [70](#_ENREF_70), [135](#_ENREF_135)^ Thus, our primary outcome measure is executive function. This study, however, extends previous exercise-cognition studies as it seeks to provide evidence of a specific effect on AD pathophysiology (i.e., disease modifying effect) of aerobic exercise on AD-related pathophysiological change in preclinical AD. Thus, we will also assess key cognitive domains that are associated with asymptomatic cerebral amyloid deposition.

CSF and imaging studies indicate that declines in global cognitive ability^[136](#_ENREF_136)^ and episodic memory (verbal-based,^[50](#_ENREF_50)^ visuospatial-based,^[137](#_ENREF_137), [138](#_ENREF_138)^ or both^[139-141](#_ENREF_139)^) are associated with the presence of cerebral amyloid. Thus, secondary outcomes include verbal and visuospatial-based episodic memory measures where we expect decline on these measures will be associated with higher amyloid deposition. Visuospatial ability is an excellent marker of preclinical AD (leading diagnosis by at least 3-years)^[142-145](#_ENREF_142)^ and was the strongest cognitive indicator for PIB-positive status in a longitudinal study on healthy aging. Visuospatial ability is also strong indicator of executive functioning ability.^[146](#_ENREF_146)^ Although studies of the cognitive effects of aerobic exercise have not resulted in major improvements in episodic memory, no studies have assessed the cognitive effects of exercise in a cohort of individuals with preclinical AD who have expected accelerated rates of decline in episodic memory. By selecting amyloid elevated individuals, we are targeting individuals with preclinical AD who may benefit most from potential disease-modifying effects of aerobic exercise on AD pathophysiology. Thus secondary outcomes include verbal and visuospatial-based episodic memory measures, where we expect decline on these measures will be associated with higher amyloid deposition.

Additionally, three reports indicate cognitive decline in preclinical AD may be mediated by structural changes in the hippocampus^[136](#_ENREF_136), [139](#_ENREF_139), [140](#_ENREF_140)^ and the concept of preclinical AD posits that hippocampal atrophy is a downstream effect of amyloid deposition. Moreover, this mediation hypothesis may explain a recent finding from Kraemer and colleagues^[147](#_ENREF_147)^ where better visual memory performance was associated with less hippocampal atrophy in exercising nondemented older adults.^[139](#_ENREF_139)^ Thus, we will test the mediation effect of hippocampal atrophy on the relationship between exercise and cognitive decline over 52-weeks across all 3 outcomes. We anticipate visual memory to be the strongest of the multiple indicators.

Data Acquisition: All participants complete a 2- 3 hour neuropsychological test battery at baseline, 26-weeks, and 52-weeks. This battery includes selected tests with demonstrated responsiveness to aerobic exercise and sensitivity to change in preclinical AD. These tests will be aggregated into several domains: Executive Function, Visual Memory, and Verbal Memory, General Health, Emotional Health.

Outcomes: Cognitive domains of Executive Function, Verbal Memory, and Visual Memory are face valid aggregates of related subtests that have generated moderate to large effect sizes across several reviews on cognition and aerobic exercise when aggregated. We have expertise using Confirmatory Factor Analysis **(CFA)** to determine the best fitting empirical model of data and create factor score weights that are sensitive indicators of cognitive change. ^[157](#_ENREF_157), [158](#_ENREF_158)^ CFA is a common method used to aggregate true score (common) variance across multiple subtests and attenuate error. Thus, factor scores yield more reliable estimates of cognitive ability than individual tests because they are purer indices of ability.^[159](#_ENREF_159), [160](#_ENREF_160)^ This is desirable because cognitive performance in aging individuals is highly variable. Individual subtests are not as efficient as CFA-based composite scores. We have shown in previous work^[142](#_ENREF_142), [161](#_ENREF_161)^ that longitudinal declines in cognitive ability can best be characterized with a model^[162](#_ENREF_162)^ of specific mental abilities.

Statistical Analyses for Aim 3**:** This intent-to-treat analysis will use a two-sample t-test to compare change from baseline to 52 weeks between our treatment and control groups on the primary (executive function) and secondary outcomes (verbal and visual memory). Secondary endpoints will be clearly indicated as such in resulting manuscripts; therefore no further multiplicity adjustments will be made. Because cognition is measured at three time points (baseline, 26-, and 52-weeks), linear mixed models will also provide additional information such as to the functional form of the potential benefits of exercise (e.g., linear increasing benefit over time, initial benefit within 26 weeks that is sustained, etc.). Assessments of these linear mixed models will be conducted by evaluating model assumptions through residual analyses (similar to that described above).

A secondary analysis will examine the longitudinal mediation^[163](#_ENREF_163)^ effect of hippocampal atrophy on the relationship between amyloid binding and cognitive decline (primary and secondary outcomes) over 52-weeks. We anticipate the relationship between exercise and improved cognition will be partially mediated by hippocampal atrophy. Briefly, to evaluate if hippocampal atrophy is a mediator, we will first assess for a positive correlation between cognition with hippocampal volume (i.e., better change in cognition measures associated with less hippocampal atrophy as measured by Pearson’s correlation coefficient, or alternatively, Spearman’s correlation coefficient if a nonparametric approach is required). We will have already evaluated the relationship between exercise and hippocampal changes in Aim 2, anticipating less atrophy in the exercise group. If these bivariate relationships demonstrate associations in these hypothesized directions, we will then model change in cognition as a function of exercise and hippocampal atrophy (simultaneously) using OLS regression. Exercise will be examined both by treatment group assignment and as a continuous measure as described above. The attenuation of the effect of exercise on cognition in these models compared to that from the models without adjustment for hippocampal atrophy, under these additional conditions, would indicate hippocampal atrophy is a mediator of the exercise-cognition relationship. Assessment of the OLS models would follow the approach described in Aim 1 (above).

**Sample Size Justification**

| **Table 2.** Anticipated study power | | | |
| --- | --- | --- | --- |
| **Study aim** | **Primary measure*** | **Effect size (Cohen’s d)** | **Power** |
| Aim 1 | Amyloid burden | 0.79 | **92.9%** |
| Aim 2 | Hippocampal volume | 0.66 | **81.4%** |
| Aim 3 | Executive function | 2.32 | **>95%** |
| *Primary measures are change from baseline to 52-week follow-up. | | | |

We have identified primary endpoints for intent-to-treat analyses between treatment groups within each study aim: change in amyloid burden (Aim 1); hippocampal atrophy (Aim 2); and change in executive function (Aim 3). Other endpoints and analyses will be described in resulting publications as secondary. For all analyses the traditional type I error rate of α=0.05 will be used. We will use two-sided tests to further protect against spurious results. Our primary intent-to-treat analyses will be based on the two-sample t-test. **Table 2** presents the power for each aim of our study corresponding to our primary measures. Anticipated effect sizes are presented using Cohen’s d (expected average group difference divided by the assumed, common standard deviation). We assumed 15% of our 100 enrolled subjects will be lost to attrition, so power calculations assumed 56 subjects in our exercise intervention arm and 28 subjects in our control group.

*Aim 1:* A 78 week study assessing Bapineuzumab^[119](#_ENREF_119)^ in AD demonstrated a mean change in cortical PIB retention (average across six cortical regions) of -0.09 (0.16 SD) for those treated with Bapineuzumab (n=20) and 0.20 (0.09) for those on placebo (n=7). Given the differences in our proposed study (preclinical AD sample, lower expected amyloid burden, shorter duration) we determined power allowing exercise to have a lower effect size. An effect size only 40% as large will produce a Cohen’s d of 0.79. Thus, Aim 1 will have 93% power to detect this conservative anticipated effect of exercise on amyloid burden (and 81.9% power to detect 30% of the Bapineuzumab effect size).

*Aim 2:* An 52-week exercise intervention study^[77](#_ENREF_77)^ found mean values of hippocampal volume of 4.89 (0.74 SD) and 4.98 (0.69) at baseline and post-intervention, respectively, in the exercise group (n=60) and 4.90 (0.80) and 4.83 (0.80), respectively, among controls (n=60). We assumed a high correlation (ρ = 0.95) for within subject baseline and post-intervention measures given our analyses of ADNI data (n=160) suggesting a within-subject r >0.99 for hippocampal volumes over 52-weeks. This resulted in an estimated effect size (Cohen’s d) of 0.66. Thus, Aim 2 will have 81% power to detect a similar effect size in hippocampal volume.

*Aim 3:* Preliminary results from our TEAM study indicate that change scores for our composite score of executive function produced a Cohen’s d of approximately 2.32 (n=53), providing >95% power to detect this effect in executive function.

*Impact of 2:1 Randomization:* We have examined the impact of our 2:1 randomization design on the power of this study and found its impact minimal. Compared to 1:1 randomization, the decrease in power for our 2:1 design is <5% for each primary measure. We believe that, in keeping with this funding mechanism (PAR-11-100) intended for pilot clinical trials, the gain in knowledge and benefits in participant recruitment from utilizing a larger experimental exercise arm outweighs this minimal loss in power, particularly given that we have sufficient power to assess our primary endpoints presented above (**Table 2**).

**Exploratory Aim: Examine potential underlying mechanisms relating exercise with brain health.**

Although the study’s primary purpose is to assess the impact of aerobic exercise on AD pathophysiology, we are collecting additional data to allow us to explore potential underlying mechanisms that may relate exercise with brain benefits. These secondary outcome measures include standard measures of physiologic adaptations to exercise (VO_2_peak, body composition, and physical function measures) and plasma based measures of BDNF, inflammation, insulin, and anabolic hormones.

*Aerobic Fitness: Peak oxygen consumption (VO_2_ peak):* VO_2_ peak is the standard, quantitative measure of aerobic fitness. VO_2_ peak will be assessed at baseline and 52-weeks during a graded treadmill exercise test to assess physiologic response to exercise and guide the exercise prescription using maximal heart rate achieved. We have extensive experience with performing VO_2_ peak in older adults^[78](#_ENREF_78), [164](#_ENREF_164)^ using the modified Bruce protocol.^[165](#_ENREF_165)^ Subjects are attached to a 12-lead electrocardiograph and wear a non-rebreathing facemask to assess heart rates, blood pressure, and expired air (oxygen, carbon dioxide) using a Parvomedics system. VO_2_ peak is considered the highest observed value during the test^[166](#_ENREF_166), [167](#_ENREF_167)^ and maximal effort is defined as meeting 3 of the 4 following criteria: a plateau in oxygen consumption, a respiratory exchange ratio (RER) ≥1.0, a maximal heart rate within 90% age-predicted maximum, or volitional fatigue.^[168](#_ENREF_168)^ For appropriate exercise prescription, adequate maximal exercise testing needs to be performed. An adequate exercise test will be defined as achieving an RER ≥1.0. Subjects not achieving an adequate exercise test will be asked to return to on a different day to repeat the test and an alternate modality may be considered. The test with the greatest VO_2_ will be used to prescribe exercise intensity.

*Body Composition:* Participants will be evaluated with dual energy x-ray absorptiometry (DEXA, Lunar Prodigy, version 11.2068, Madison, WI) to determine fat-free mass, fat mass, and percent body fat at baseline, and 52 weeks. DEXA uses very low X-ray doses (0.02mREM) to detect changes in body composition on the order of 1.6-3.8%.^[169](#_ENREF_169), [170](#_ENREF_170)^ Body composition is also assessed with standard anthropometric measures including body weight using a digital scale accurate to ±0.1 kg (Seca Platform Scale, model 707, Seca Corp.) prior to breakfast and after attempting to void. Circumference measurements are taken with a Gullick II circumference measurement tape at the smallest girth around the trunk and at the widest protrusion of the buttocks.

*Physical function measures:* We will complete an efficient battery of functional assessments indexing endurance, strength, balance, and flexibility at baseline and 52 weeks to assess physiologic and functional adaptations to exercise. The *Physical Performance Test* ^[171](#_ENREF_171)^ is a short, objective battery of timed physical tasks necessary for daily self-care that is commonly used in exercise trials,^[172](#_ENREF_172)^ including our own.^[78](#_ENREF_78), [164](#_ENREF_164)^ The *Senior Fitness Test*^[173](#_ENREF_173)^ is a 7-item battery of measures including flexibility, strength and endurance that is sensitive to change with exercise training.^[174](#_ENREF_174)^ *Grip strength* is associated with functional independence^[175](#_ENREF_175)^ and will be measured in the dominant hand using a grip dynamometer (JAMAR, Sammons Preston, Bolingbrook, IL) as an average of 3 maximal grips. *Lower extremity strength* will be assessed as the average of 3 forceful knee extensions with a hand held dynamometer (MicroFET, Hoggan, IN). The *Late-Life Functional Disability Index*^[176](#_ENREF_176), [177](#_ENREF_177)^ assesses disability and function across a variety of life tasks and is reliable measure of change in function with exercise training.^[178](#_ENREF_178), [179](#_ENREF_179)^

*Laboratory assays and blood banking:* Phlebotomy will be performed at baseline and after 52 weeks of exercise. Serum, plasma, DNA, and platelets will be banked in the Biospecimen Shared Resource (BSR). The BSR is the main repository for all KU ADC specimens. The concentrations of insulin, BDNF, IGF-I, TNF-α, C-reactive protein, and IL-6 will be measured using enzyme-linked immunosorbent assay kits (ELISA).

Exploratory Aim Statistical Analyses: Our exploratory aim will assess potential mechanisms relating 52-weeks of aerobic exercise to brain health. We will examine if aerobic exercise results in changes in body composition and anabolic and inflammatory markers. We will first assess changes over one year in body composition (fat and lean mass, BMI) and serum markers (BDNF, insulin sensitivity, IGF-1, TNF-alpha, CRP, IL-6) by comparing the aerobic and nonaerobic groups using methods described in aim 1 (two-sample t-test with assessment of assumptions). We suspect the aerobic exercise group will have changes in body composition (increased lean mass, reductions in fat mass), increased BDNF, reduced inflammatory markers, and enhanced insulin sensitivity. Next, we will explore if change in these measures predict 52-week change in amyloid burden, brain atrophy, and cognition using OLS models and assessment as described in aim 1.

**Data management**

*Clinical Data Management System:* Study data will be collected and managed using REDCap (Research Electronic Data Capture) web-based, electronic data capture tools hosted on a secure, HIPAA compliant server at KUMC.^[180](#_ENREF_180)^ REDCap provides 1) validated data entry; 2) audit trails; 3) automated export 4) data import tools. We have extensive experience developing, maintaining and using REDCap project structures for the KU ADC and our TEAM studies. The proposed project will capitalize on this previous work.

*Data Collection Forms:* All data will be collected on standard source documents and entered into standard case report forms in REDCap. These forms have been developed and are currently in use to support data collection for ongoing exercise interventions and to support the KU ADC clinical and cognitive evaluations. Forms include daily exercise data forms used by the exercise instructors and paper and electronic source documents for outcome and clinical assessments. The Department of Biostatistics ensures data security by managing all data on a secure server that has role-based access that is password protected. All files that are modified are backed up daily, with complete backups of the server on a weekly basis. All data are stored in a HIPAA compliant manner.

**Protection of Human Subjects**

**Risks to the Subjects**

***Human Subjects Involvement and Characteristics:*** We plan to enroll 120 subjects from a screening population of 400. Subjects are recruited from the greater metropolitan Kansas City area (population 1.8 million).

Inclusion criteria include

- Preclinical AD (CDR 0 [nondemented] with evidence of cerebral amyloidosis)
- Non-elevated Florbetapir PET scan algorithm computerized measuring a mean quantitative standard uptake value ratio equal to or greater than 1.0 for 6 regions of interest.
- Age 65 or older
- Sedentary or underactive by the Telephone Assessment of Physical Activity
- Stable doses of medications for 30 days.
- Clinician judgement regarding subject’s health status and likelihood to successfully complete the 1-year exercise intervention

Exclusion criteria include

- Clinically significant major psychiatric disorder (e.g., Major Depressive Disorder) according to DSM-IV criteria or significant psychiatric symptoms (e.g., hallucinations) that could impair the completion of the study
- Clinically-significant systemic illness likely to result in deterioration of the patient’s condition or affect the patient’s safety during the study
- History of clinically-evident stroke
- Clinically-significant infection within the last 30 days
- Active cardiac condition (e.g. angina, myocardial infarction, atrial fibrillation) or pulmonary condition in the past 2 years that, in the investigator’s opinion, could pose a safety risk to the pariticipant—unless cleared for exercise by the participant’s primary care physician or cardiologist.
- Uncontrolled hypertension within the last 6 months
- History of cancer within the last 5 years (except non-metastatic basal or squamous cell carcinoma)
- History of drug or alcohol abuse as defined by DSM-IV criteria within the last 2 years
- Insulin-dependent diabetes mellitus
- Significant pain or musculoskeletal disorder that would prohibit participation in an exercise program
- Unwillingness to undergo or contraindication to brain MRI scan.
- History within the last 5 years of primary or recurrent malignant disease with the exception of resected localized cutaneous squamous cell carcinoma, basal cell carcinoma, cervical carcinoma, or prostate cancer.

***Sources of Materials:*** Research material obtained from participants consists of clinical and behavioral data. This includes information from a subject and collateral source interview, physical / neurological examination, and neuropsychological testing. Blood specimens are obtained for laboratory studies. Data on body composition (fat free mass, fat mass, bone mass) and peak oxygen consumption as well as mood and quality of life will be obtained. These data are used for research purposes only and specific individual results are not provided to subjects and their families. There is no billing of subjects, their families, or third party payers for the research assessments.

***Potential risks:*** The clinical research procedures consist of history taking, physical / neurological examination, and psychometric testing. Embarrassment could occur should the subjects believe they answer questions incorrectly.

DEXA scanning involves low doses of radiation, equivalent to 0.1 mrem while the typical radiation exposure from a normal chest x-ray is 30 mrem.

Risks involving laboratory testing include minor pain, bruising, and swelling at the needle site, discomfort of hunger or thirst due to fasting, and less likely is the possibility of lightheadedness, or even briefly feeling faint during the blood drawing procedure. Neuroimaging is associated with risk of anxiety or embedded metal displacement.

Measurement of peak oxygen consumption involves a graded exercise test. Potential risks of the exercise test as well as the exercise intervention include unpredictable changes in blood pressure or heart rhythm, myocardial infarction and death as well as the less serious problems of fatigue, muscle soreness, tripping, falling, and other injury to tendons, ligaments, joints, and muscles. There is no statistical information regarding serious complications during exercise testing in patients without ischemic coronary heart disease. A review of the experience of 30 cardiac rehabilitation programs reported information on 13,750 participants who accumulated 1,629,634 patient hours of supervised exercise. A total of 50 cardiac arrests were observed, 42 of which were successfully resuscitated while 8 were fatal.([Haskell 1978](#_ENREF_4))

Randomized trials of exercise training in individuals with ischemic heart disease have shown that mortality is lower in patients who exercise than those that do not.([Froelicher, Jensen et al. 1985](#_ENREF_3); [Belardinelli, Georgiou et al. 1999](#_ENREF_2)) It seems reasonable to assume that individuals without recent (> 2 years) clinical evidence of ischemic heart disease will have a lower incidence of cardiovascular complications during medically supervised exercise testing than individuals with ischemic heart disease.

Additionally, there are certain risks and discomforts that may be associated with exercise that include temporary shortness of breath, muscle fatigue, sweating, and physical discomfort. Muscles may be sore up to three days after performing the exercise. Also, there exists the possibility of an undiagnosed medical problem that may surface during the exercise. During testing, participants will be carefully observed by members of the research team to reduce these risks associated with exercise.

***Adverse Events:*** Adverse events will be defined as any untoward medical occurrence in study participants or others immediately involved in the performance of the protocol, which does not necessarily have a causal relationship with the study treatment, but results in a change in intervention, daily function, hospitalization or rated category 3 or above using the National Cancer Institute Common Terminology Criteria for Adverse Events (CTCAE) v3.0.^209^ Expected events such as slight muscle soreness consistent with exercise, or those consistent with a participant’s prior medical history not sufficient to alter the intervention will not be considered an AE. Staff involved in performance of the protocol (e.g. trainers, dieticians, coordinators) will continually monitor participants for adverse events throughout the intervention, and study staff will assess adverse events at every testing visit as well as during regularly scheduled telephone assessments with all randomized participants.

If study staff, tester, participant, caregiver or exercise instructor reports adverse events or complaints, relevant information will be collected and documented.

The participant will be evaluated by the unblinded investigator identified in the protocol. The investigator will have access to training logs, telephone assessment forms, and all study-related testing results. The investigator will determine the severity (according to the CTCAE) and relatedness of the AE to the intervention. The investigator will identify adverse events of clinical concern, those that may require further workup, or suggest that additional participation in the intervention might be a safety risk. These AEs will first be discussed with the participant and then communicated to the study staff or providers with participant’s consent and as appropriate.

All maximal exercise testing will be monitored by an appropriate medical monitor.If the subject has performed a maximal exercise test in the past, we will, with the participant’s consent, request the records from the testing to use as baseline ECG. Incidental research findings noted by the medical monitor (e.g. ECG abnormalities) will be discussed with the participant. Participants will be referred to a provider as requested. Imaging (e.g. MR, DXA) will be formally read only when required by the protocol and as outlined to the participant in the consent form. All adverse events will be reported to the investigator, study coordinator, the Data Safety Monitoring Board and the Human Subjects Committee per KUMC Human Subject Committee reporting policies.

.

**Adequacy of Protection Against Risk**

***Recruitment and Informed Consent:*** Recruitment occurs by referrals from organizations serving large numbers of older adults, public service announcements through media, physician referral, and word of mouth.

We are very sensitive to issues related to the subject’s ability to understand the elements of consent and their capacity to make decisions. Individuals with dementia will be excluded from the study. Informed consent will be obtained by study staff (as the designee for Dr. Burns); the purposes of the study, its assessment procedures, and risks and benefits are explained. The original signed consent form is kept with the subject’s confidential file and a copy is given to the subject.

***Protection Against Risk:*** All research data are maintained confidentially by numerical code in password-protected databases. All paper copies are filed by number in accordance with professional standards of privileged information. Confidentiality is strictly safeguarded by HIPAA-compliant standards.

Empathetic and professional staff mitigates risk of embarrassment. An experienced nurse clinician performs venipuncture. All women in the study will be post-menopausal and thus not at risk of pregnancy, mitigating the risks of low dose radiation from DEXA scanning. The risk from radiation exposure of this magnitude is too small to be measured directly and is considered to be negligible when compared with other everyday risk. Risk from MRI is mitigated by trained technicians who thoroughly screen participants for exclusionary conditions prior to each MRI session.

To protect against and minimize potential risks during the assessment of VO_2_ peak, subjects will be carefully evaluated during the clinical assessment with a physical examination and a resting ECG. The subjects will be screened to exclude those with a history of ischemic heart disease. During the exercise test, a physician will be present and monitor the subject’s heart rhythm by electrocardiography and blood pressure. American College of Sports Medicine (ACSM) guidelines will be used to determine whether the exercise test should be terminated early. Criteria for immediate termination include chest discomfort, ST-segment depression (>2mm), nervous system symptoms (i.e., ataxia, dizziness), and sustained ventricular tachycardia.

Risks for the treadmill exercise intervention are similar to those of exercise testing as detailed above. Participants will be initially supervised by a fitness specialist to ensure safety. Kill switches and handrails are standard on the treadmills. The program will begin with light exercise and progressively increase in intensity. Sessions are supervised by a trained professional who will focus on avoiding injuries and adapting the program to participants’ needs. If subjects develop chest discomfort, other symptoms (for example, dizziness), or injury, the session will be discontinued immediately.

***Florbetapir PET Results Disclosure:*** The KU ADC is disclosing results of the Florbetapir PET scans to participants. This is done under a separate consent to the exercise trial outlined in this proposal although we are including information here regarding the rationale for disclosure and how risks will be mitigated.

With the advent of molecular imaging, there is an increasing interest in the use of AD biomarkers in diagnosis and risk assessment. As of February 2011, these techniques are confined to the research arena although with the expected approval of agents by the FDA in the near future, their use will likely soon become widespread in clinical use. The KU ADC is at the forefront in this biomarker research and the KU ADC’s Florbetapir substudy creates important opportunities that allow us to participate in and shape the national conversation on how AD biomarkers are used for risk assessment. Additionally, identifying a cohort of individuals with and without asymptomatic cerebral amyloidosis is designed to provide the essential foundation for performing necessary rigorous studies assessing AD prevention strategies.

In our review of the literature, we believe our choice to reveal amyloid status has several advantages and relatively low risks to the participants. Hallmark studies in this area (Roberts et al., 2005; Carpenter et al., 2008) indicate that most people want to know if they have a specific diagnosis (risk for AD or the diagnosis; Bamford et al. 2004; Carpenter & Dave, 2004). In these studies, reports of anxiety and depression were actually higher before diagnosis than after, and low reporting of depressive symptoms persisted throughout the 6-week, 6-month and 1-year follow up periods. Thus, the overwhelming majority of people who received news of their elevated risk profile or a diagnosis of AD did not experience grief, depression, or anxiety as a result of that disclosure.  Thus “bad news” does not necessarily lead to a catastrophic emotional reaction (Aminzadeh et al., 2007; Carpenter et al., 2008)

Although we do not anticipate many participants to experience reactive depression or anxiety in response to amyloid status disclosure, we are putting in place safety monitoring procedures to detect those individuals who do experience distress and provide mental health referrals when appropriate. Based on the literature (Roberts et al., 2005; Carpenter, 2009; Green et al., 2009), we anticipate 5 categories of possible reaction that need to be monitored: relief, depressive reaction, anger, anxiety, or a blend of these four categories. Negative reactions were more likely to occur when the participant is male, lower educated, and young (below age 60). Knowing these are demographic characteristics will help the clinical research team to expedite the identification of individuals experiencing distress. In the current study we include the identical measures used in the hallmark studies to insure that we are using best-practices to monitor for adverse reaction to disclosure of asymptomatic cerebral amyloidosis.

***Non-elevated Amyloid Group:*** We will recruit individuals with non-elevated amyloid PET scans who have a mean SUVR (in 6 regions of interest) of 1.0 and greater to be enrolled into the trial as part of the non-elevated amyloid group. Based on our first 84 non-elevated participants screened for the study, this represents about 1/3^rd^ of those with non-elevated amyloid PET scans. Participants will be recruited by letter or phone calls to those who have been previously screened and meet the criteria or during in-person screening visits until a total of 50 nonelevated participants are enrolled.

SUVR (Standard Uptake Value Ratio) represents a ratio of the retention of radiotracer in the brain compared to the patient’s control region of the cerebellum. A higher is believed to reflect higher levels of amyloid in the brain although in this group of individuals who do not evidence of amyloid deposition given their non-elevated status, the relevance of the SUVR value remains uncertain. Nevertheless, this group is of scientific interest given some early data suggesting the group may be enriched with individuals who accumulate amyloid over time.

Rationale for not disclosing SUVR values: Participants will be informed that they are in a group representing 1/3^rd^ of those with non-elevated scans that have been selected as eligible to participate based on computer algorithm. Our current practice is not to communicate information on patient’s SUVR values and we believe this continues to be important until more data is available to suggest that SUVR values are shown to be clinically relevant. There is currently no consensus or evidence that within non-elevated participants SUVR values have clinical relevance. It remains unclear that an SUVR value, especially in the non-elevated range, provides any predictive information on risk. Additionally, it remains unclear whether the sensitivity and precision of the measure influences how these values can be interpreted. Not disclosing SUVR values to participants is the current practice for the national Alzheimer’s Disease Neuroimaging Initiative. We have a scientific interest in the group of individuals in the top third of SUVR measures as there is early evidence that those with higher amyloid burdens may accumulate amyloid at a more rapid rate than others. Whether individuals who accumulate amyloid are at higher risk of cognitive change is not yet known although given that our primary aim is to assess the impact of aerobic exercise on amyloid accumulation.

Given the burden and expense of amyloid scanning, MRI scanning, and a one-year exercise program, it is not feasible to enroll a sample of all non-elevated participants.

**Potential Benefits of the Proposed Research to the Subjects and Others**

Subjects potentially benefit from participation in a supervised exercise program.

**Importance of the Knowledge to be Gained**

Society benefits from research advances in aging and the maintenance of cognitive and brain health. Improved fitness through supervised rehabilitation programs may represent an important therapeutic or preventive strategy for dementia. This study will enhance our understanding of the role of exercise in promoting healthy brain aging. Potential benefits outweigh the minimal risks of this research.

**Data Safety and Monitoring Plan**

An internal safety committee will meet quarterly to review adverse events and the overall safety of the ongoing trial. The committee will be composed of three clinicians: Mamatha Pasnoor, MD, Richard Dubinsky, MD, and James Vacek, MD. The primary responsibilities of the DSMB are to 1) periodically review and evaluate the accumulated study data for participant safety, study conduct and progress, and efficacy when planned in the protocol , and 2) make recommendations to the PI concerning the continuation, modification, or termination of the trial.

Should a study participant experience an AE, relevant information will be collected and documented, and the AE will be reviewed by the appropriate investigator within 24 hours. If the investigator determines the AE is directly related to the study, raises concern about increased risk to the participant or overall safety of the study design, the safety committee will be notified within 24 hours. The DSMB will evaluate the AE and will make recommendations based on the protocol (e.g. stopping rules).

**Women and Minority Inclusion in Clinical Research**

***Inclusion of women:*** Women are included in all studies described. We expect that approximately 50% of the subjects will be women.

***Inclusion of minorities:*** All minority groups are encouraged to participate in this and all our research projects. We place an extra emphasis on the recruitment of minorities through the KU ADC and the Landon Center on Aging. The ADC Education Core recently hired a minority outreach coordinator to increase enrollment of African Americans into ADC studies. The Landon Center on Aging community outreach effort specifically targets the Hispanic and African American populations through activity in service agencies for these populations and with targeted advertising in minority publications. In 2003, the University of Kansas Medical Center’s outpatient population was composed of 18% African-American, 10% Hispanic, 1% Asian, and 3% other. We expect this proportion of minorities will be reflected in our enrollment. All minority groups are encouraged to participate in the research projects.

REFERENCES

1. Middleton LE, Yaffe K. Targets for the prevention of dementia. J Alzheimers Dis;20:915-924.

2. Kramer AF, Hahn S, Cohen NJ, et al. Ageing, fitness and neurocognitive function. Nature 1999;400:418-419.

3. Colcombe S, Kramer AF. Fitness effects on the cognitive function of older adults: A meta-analytic study. Psychological Science 2003;14:125-130.

4. Friedland RP, Fritsch T, Smyth KA, et al. Midlife recreational activity inversely associated with Alzheimer's disease risk. Proc Natl Acad Sci 2001;98:3440-3445.

5. Laurin D, Verreault R, Lindsay J, MacPherson K, Rockwood K. Physical activity and risk of cognitive impairment and dementia in elderly persons. Arch Neurol 2001;58:498-504.

6. Yaffe K, Barnes D, Nevitt M, Lui LY, Covinsky K. A Prospective Study of Physical Activity and Cognitive Decline in Elderly Women: Women Who Walk. Archives of Internal Medicine 2001;161:1703-1708.

7. Pignatti F, Rozzini R, Trabucchi M, Yaffe K. Physical Activity and Cognitive Decline in Elderly Persons. Archives of Internal Medicine 2002;162:361-362.

8. Dustman RE, Ruhling RO, Russell EM, et al. Aerobic Exercise Training and Improved Neuropsychological Function of Older Individuals. Neurobiology of aging 1984;5:35-42.

9. Hassmen P, Koivula N. Mood, physical working capacity and cognitive performance in the elderly as related to physical activity. Aging-Clinical and Experimental Research 1997;9:136-142.

10. Williams P, Lord SR. Effects of group exercise on cognitive functioning and mood in older women. Australian and New Zealand Journal of Public Health 1997;21:45-52.

11. Hill RD, Storandt M, Malley M. The impact of long-term exercise training on psychological function in older adults. J Gerontol 1993;48:12-17.

12. Barnes DE, Yaffe K, Satariano WA, Tager IB. A Longitudinal Study of Cardiorespiratory Fitness and Cognitive Function in Healthy Older Adults. Journal of the American Geriatrics Society 2003;51:459-465.

13. Weuve J, Kang JH, Manson JE, Breteler MMB, Ware JH, Grodstein F. Physical Activity, Including Walking, and Cognitive Function in Older Women. JAMA: The Journal of the American Medical Association 2004;292:1454-1461.

14. Abbott RD, White LR, Ross GW, Masaki KH, Curb JD, Petrovitch H. Walking and Dementia in Physically Capable Elderly Men. JAMA: The Journal of the American Medical Association 2004;292:1447-1453.

15. Adlard PA, Perreau VM, Pop V, Cotman CW. Voluntary Exercise Decreases Amyloid Load in a Transgenic Model of Alzheimer's Disease. Journal of Neuroscience 2005;25:4217-4221.

16. Nation DA, Hong S, Jak AJ, et al. Stress, exercise, and Alzheimer's disease: a neurovascular pathway. Med Hypotheses;76:847-854.

17. Radak Z, Hart N, Sarga L, et al. Exercise plays a preventive role against Alzheimer's disease. J Alzheimers Dis;20:777-783.

18. Ke HC, Huang HJ, Liang KC, Hsieh-Li HM. Selective improvement of cognitive function in adult and aged APP/PS1 transgenic mice by continuous non-shock treadmill exercise. Brain Res;1403:1-11.

19. Leem YH, Lee YI, Son HJ, Lee SH. Chronic exercise ameliorates the neuroinflammation in mice carrying NSE/htau23. Biochem Biophys Res Commun;406:359-365.

20. Burns JM, Cronk BB, Anderson HS, et al. Cardiorespiratory fitness and brain atrophy in early Alzheimer disease. In, 2008: 210-216.

21. Honea RA, Thomas GP, Harsha A, et al. Cardiorespiratory fitness and preserved medial temporal lobe volume in Alzheimer disease. Alzheimer Dis Assoc Disord 2009;23:188-197.

22. Aisen PS, Andrieu S, Sampaio C, et al. Report of the task force on designing clinical trials in early (predementia) AD. Neurology 2011;76:280-286.

23. Burns JM, Johnson DK, Watts A, Swerdlow RH, Brooks WM. Reduced lean mass in early Alzheimer disease and its association with brain atrophy. Arch Neurol 2010;67:428-433.

24. Cronk BB, Johnson DK, Burns JM. Body mass index and cognitive decline in mild cognitive impairment. Alzheimer Dis Assoc Disord 2010;24:126-130.

25. Loskutova N, Honea RA, Brooks WM, Burns JM. Reduced limbic and hypothalamic volumes correlate with bone density in early Alzheimer's disease. J Alzheimers Dis 2010;20:313-322.

26. Loskutova N, Honea RA, Vidoni ED, Brooks WM, Burns JM. Bone density and brain atrophy in early Alzheimer's disease. J Alzheimers Dis 2009;18:777-785.

27. Statistics FIFoA-R. Older Americans 2008: Key Indicators of Well-Being. Washington, DC, 2009.

28. Prigerson HG. Costs to Society of Family Caregiving for Patients with End-Stage Alzheimer's Disease. The New England Journal of Medicine 2003;349:1891-1892.

29. Aging NIo. Growing Older in America: the Healthy and Retirement Study. Bethesda, MD: National Institutes of Health, 2007.

30. Sperling RA, Aisen PS, Beckett LA, et al. Toward defining the preclinical stages of Alzheimer's disease: Recommendations from the National Institute on Aging-Alzheimer's Association workgroups on diagnostic guidelines for Alzheimer's disease. Alzheimers Dement 2011;7:280-292.

31. Desikan RS, McEvoy LK, Thompson WK, et al. Amyloid-beta associated volume loss occurs only in the presence of phospho-tau. Ann Neurol;70:657-661.

32. Jack CR, Jr., Knopman DS, Jagust WJ, et al. Hypothetical model of dynamic biomarkers of the Alzheimer's pathological cascade. Lancet Neurol 2010;9:119-128.

33. Clark CM, Schneider JA, Bedell BJ, et al. Use of Florbetapir-PET for Imaging Î²-Amyloid Pathology. JAMA: The Journal of the American Medical Association;305:275-283.

34. Price JL, Morrism J.C. Tangles and plaques in nondemented aging and "preclinical" Alzheimer's disease. . Annals of Neurology 1999;45:358-368.

35. Price JL, McKeel DW, Jr., Buckles VD, et al. Neuropathology of nondemented aging: presumptive evidence for preclinical Alzheimer disease. Neurobiology of aging 2009;30:1026-1036.

36. Bennett DA, Schneider JA, Arvanitakis Z, et al. Neuropathology of older persons without cognitive impairment from two community-based studies. Neurology 2006;66:1837-1844.

37. Shaw LM, Vanderstichele H, Knapik-Czajka M, et al. Cerebrospinal fluid biomarker signature in Alzheimer's disease neuroimaging initiative subjects. Ann Neurol 2009;65:403-413.

38. Fleisher AS, Chen K, Liu X, et al. Using positron emission tomography and florbetapir F18 to image cortical amyloid in patients with mild cognitive impairment or dementia due to Alzheimer disease. Arch Neurol;68:1404-1411.

39. Storandt M, Mintun MA, Head D, Morris JC. Cognitive decline and brain volume loss as signatures of cerebral amyloid-beta peptide deposition identified with Pittsburgh compound B: cognitive decline associated with Abeta deposition. Arch Neurol 2009;66:1476-1481.

40. Mintun MA, LaRossa GN, Sheline YI, et al. [11C]PIB in a nondemented population: Potential antecedent marker of Alzheimer disease. Neurology 2006;67:446-452.

41. Aizenstein HJ, Nebes RD, Saxton JA, et al. Frequent amyloid deposition without significant cognitive impairment among the elderly. Arch Neurol 2008;65:1509-1517.

42. Jack CR, Jr., Wiste HJ, Vemuri P, et al. Brain beta-amyloid measures and magnetic resonance imaging atrophy both predict time-to-progression from mild cognitive impairment to Alzheimer's disease. Brain 2010;133:3336-3348.

43. Koivunen J, Scheinin N, Virta JR, et al. Amyloid PET imaging in patients with mild cognitive impairment: a 2-year follow-up study. Neurology;76:1085-1090.

44. Ewers M, Insel P, Jagust WJ, et al. CSF Biomarker and PIB-PET-Derived Beta-Amyloid Signature Predicts Metabolic, Gray Matter, and Cognitive Changes in Nondemented Subjects. Cereb Cortex.

45. Okonkwo OC, Mielke MM, Griffith HR, et al. Cerebrospinal fluid profiles and prospective course and outcome in patients with amnestic mild cognitive impairment. Arch Neurol 2011;68:113-119.

46. Morris JC, Roe CM, Grant EA, et al. Pittsburgh Compound B Imaging and Prediction of Progression From Cognitive Normality to Symptomatic Alzheimer Disease. Arch Neurol 2009;66:1469-1475.

47. Rentz DM, Locascio JJ, Becker JA, et al. Cognition, reserve, and amyloid deposition in normal aging. Ann Neurol;67:353-364.

48. Mormino EC, Kluth JT, Madison CM, et al. Episodic memory loss is related to hippocampal-mediated beta-amyloid deposition in elderly subjects. Brain 2009;132:1310-1323.

49. Stomrud E, Hansson O, Zetterberg H, Blennow K, Minthon L, Londos E. Correlation of longitudinal cerebrospinal fluid biomarkers with cognitive decline in healthy older adults. Arch Neurol;67:217-223.

50. Resnick SM, Sojkova J, Zhou Y, et al. Longitudinal cognitive decline is associated with fibrillar amyloid-beta measured by [11C]PiB. Neurology;74:807-815.

51. Okonkwo OC, Alosco ML, Griffith HR, et al. Cerebrospinal fluid abnormalities and rate of decline in everyday function across the dementia spectrum: normal aging, mild cognitive impairment, and Alzheimer disease. Arch Neurol;67:688-696.

52. Fjell AM, Walhovd KB, Fennema-Notestine C, et al. Brain atrophy in healthy aging is related to CSF levels of Abeta1-42. Cereb Cortex;20:2069-2079.

53. Jack CR, Jr., Lowe VJ, Senjem ML, et al. 11C PiB and structural MRI provide complementary information in imaging of Alzheimer's disease and amnestic mild cognitive impairment. Brain 2008;131:665-680.

54. Jack CR, Jr., Lowe VJ, Weigand SD, et al. Serial PIB and MRI in normal, mild cognitive impairment and Alzheimer's disease: implications for sequence of pathological events in Alzheimer's disease. Brain 2009;132:1355-1365.

55. Archer HA, Edison P, Brooks DJ, et al. Amyloid load and cerebral atrophy in Alzheimer's disease: an 11C-PIB positron emission tomography study. Ann Neurol 2006;60:145-147.

56. Becker JA, Hedden T, Carmasin J, et al. Amyloid-beta associated cortical thinning in clinically normal elderly. Ann Neurol;69:1032-1042.

57. Chetelat G, Villemagne VL, Pike KE, et al. Larger temporal volume in elderly with high versus low beta-amyloid deposition. Brain;133:3349-3358.

58. Dickerson BC, Bakkour A, Salat DH, et al. The cortical signature of Alzheimer's disease: regionally specific cortical thinning relates to symptom severity in very mild to mild AD dementia and is detectable in asymptomatic amyloid-positive individuals. Cereb Cortex 2009;19:497-510.

59. Bourgeat P, Chetelat G, Villemagne VL, et al. Beta-amyloid burden in the temporal neocortex is related to hippocampal atrophy in elderly subjects without dementia. Neurology;74:121-127.

60. Oh H, Mormino EC, Madison C, Hayenga A, Smiljic A, Jagust WJ. beta-Amyloid affects frontal and posterior brain networks in normal aging. Neuroimage;54:1887-1895.

61. Sperling RA, Laviolette PS, O'Keefe K, et al. Amyloid deposition is associated with impaired default network function in older persons without dementia. Neuron 2009;63:178-188.

62. Hedden T, Van Dijk KR, Becker JA, et al. Disruption of functional connectivity in clinically normal older adults harboring amyloid burden. J Neurosci 2009;29:12686-12694.

63. Sheline YI, Raichle ME, Snyder AZ, et al. Amyloid plaques disrupt resting state default mode network connectivity in cognitively normal elderly. Biological psychiatry 2010;67:584-587.

64. Blair SN, Kampert JB, Kohl HW, III, et al. Influences of cardiorespiratory fitness and other precursors on cardiovascular disease and all-cause mortality in men and women. JAMA: The Journal of the American Medical Association 1996;276:205-210.

65. Lakka TA, Laukkanen JA, Rauramaa R, et al. Cardiorespiratory Fitness and the Progression of Carotid Atherosclerosis in Middle-Aged Men. Annals of Internal Medicine 2001;134:12-20.

66. Kurl S, Laukkanen JA, Rauramaa R, Lakka TA, Sivenius J, Salonen JT. Cardiorespiratory Fitness and the Risk for Stroke in Men. Archives of Internal Medicine 2003;163:1682-1688.

67. Sandvik L, Erikssen J, Thaulow E, Erikssen G, Mundal R, Rodahl K. Physical Fitness as a Predictor of Mortality among Healthy, Middle-Aged Norwegian Men. The New England Journal of Medicine 1993;328:533-537.

68. Dustman RE, Emmerson RY, Ruhling RO, et al. Age and fitness effects on EEG, ERPs, visual sensitivity, and cognition. NeurobiolAging 1990;11:193-200.

69. van Boxtel MP, Paas FG, Houx PJ, Adam JJ, Teeken JC, Jolles J. Aerobic capacity and cognitive performance in a cross-sectional aging study. Med Sci Sports Exerc 1997;29:1357-1365.

70. Shay KA, Roth DL. Association Between Aerobic Fitness and Visuospatial Performance in Healthy Older Adults. Psychology and Aging 1992;7:15-24.

71. Friedland RP, Fritsch T, Smyth KA, et al. Patients with Alzheimer's disease have reduced activities in midlife compared with healthy control-group members. Proceedings of the National Academy of Sciences 2001;98:3440-3445.

72. Albert MS, Jones K, Savage CR, et al. Predictors of cognitive change in older persons: MacArthur studies of successful aging. Psychol Aging 1995;10:578-589.

73. Larson EB, Wang L, Bowen JD, et al. Exercise Is Associated with Reduced Risk for Incident Dementia among Persons 65 Years of Age and Older. Ann Intern Med 2006;144:73-81.

74. Vidoni ED, Honea RA, Billinger SA, Swerdlow RH, Burns JM. Cardiorespiratory fitness is associated with atrophy in Alzheimer's and aging over 2 years. Neurobiology of aging 2011.

75. Colcombe SJ, Erickson KI, Raz N, et al. Aerobic Fitness Reduces Brain Tissue Loss in Aging Humans. Journals of Gerontology Series A: Biological Sciences and Medical Sciences 2003;58:M176-M180.

76. Colcombe SJ, Kramer AF, Erickson KI, et al. Cardiovascular fitness, cortical plasticity, and aging. Proceedings of the National Academy of Sciences 2004;101:3316-3321.

77. Erickson KI, Voss MW, Prakash RS, et al. Exercise training increases size of hippocampus and improves memory. Proc Natl Acad Sci U S A 2011;108:3017-3022.

78. Burns JM, Donnelly JE, Anderson HS, et al. Cardiorespiratory Fitness and Brain Atrophy in Early Alzheimer's Disease. Neurology 2008;In Press.

79. Honea RA, Thomas GP, Harsha A, et al. Cardiorespiratory fitness and preserved medial temporal lobe volume in Alzheimer’s Disease. Alzheimer Dis Assoc Disord 2009;*In Press*.

80. Johnson DK, Wilkins CH, Morris JC. Accelerated Weight Loss May Precede Diagnosis in Alzheimer Disease. Arch Neurol 2006;63:1312-1317.

81. Vidoni ED, Townley RA, Honea RA, Burns JM. Alzheimer disease biomarkers are associated with body mass index. Neurology;77:1913-1920.

82. Nichol K, Deeny SP, Seif J, Camaclang K, Cotman CW. Exercise improves cognition and hippocampal plasticity in APOE epsilon4 mice. Alzheimers Dement 2009;5:287-294.

83. Garcia-Mesa Y, Lopez-Ramos JC, Gimenez-Llort L, et al. Physical exercise protects against Alzheimer's disease in 3xTg-AD mice. J Alzheimers Dis;24:421-454.

84. van Praag H, Christie BR, Sejnowski TJ, Gage FH. Running enhances neurogenesis, learning, and long-term potentiation in mice. Proc Natl Acad Sci U S A 1999;96:13427-13431.

85. Barde YA. Neurotrophins: a family of proteins supporting the survival of neurons. ProgClin Biol Res 1994;390:45-56.

86. Stummer W, Weber K, Tranmer B, Baethmann A, Kempski O. Reduced mortality and brain damage after locomotor activity in gerbil forebrain ischemia. Stroke; a journal of cerebral circulation 1994;25:1862-1869.

87. Carro E, Trejo JL, Busiguina S, Torres-Aleman I. Circulating insulin-like growth factor I mediates the protective effects of physical exercise against brain insults of different etiology and anatomy. The Journal Of Neuroscience: The Official Journal Of The Society For Neuroscience 2001;21:5678-5684.

88. Lu B, Chow A. Neurotrophins and hippocampal synaptic transmission and plasticity. J Neurosci Res 1999;58:76-87.

89. Black JE, Isaacs KR, Anderson BJ, Alcantara AA, Greenough WT. Learning causes synaptogenesis, whereas motor activity causes angiogenesis, in cerebellar cortex of adult rats. Proceedings of the National Academy of Sciences of the United States of America 1990;87:5568-5572.

90. Isaacs KR, Anderson BJ, Alcantara AA, Black JE, Greenough WT. Exercise and the brain: angiogenesis in the adult rat cerebellum after vigorous physical activity and motor skill learning. Journal Of Cerebral Blood Flow And Metabolism: Official Journal Of The International Society Of Cerebral Blood Flow And Metabolism 1992;12:110-119.

91. Seals DR, Hagberg JM, Hurley BF, Ehsani AA, Holloszy JO. Effects of endurance training on glucose tolerance and plasma lipid levels in older men and women. JAMA: The Journal of the American Medical Association 1984;252:645-649.

92. Hughes VA, Fiatarone MA, Fielding RA, et al. Exercise increases muscle GLUT-4 levels and insulin action in subjects with impaired glucose tolerance. Am J Physiol 1993;264:E855-E862.

93. Kirwan JP, Kohrt WM, Wojta DM, Bourey RE, Holloszy JO. Endurance exercise training reduces glucose-stimulated insulin levels in 60- to 70-year-old men and women. J Gerontol 1993;48:M84-M90.

94. Cox JH, Cortright RN, Dohm GL, Houmard JA. Effect of aging on response to exercise training in humans: skeletal muscle GLUT-4 and insulin sensitivity. Journal of Applied Physiology 1999;86:2019-2025.

95. Kahn SE, Larson VG, Beard JC, et al. Effect of exercise on insulin action, glucose tolerance, and insulin secretion in aging. Am J Physiol 1990;258:E937-E943.

96. Houmard JA, Tyndall GL, Midyette JB, et al. Effect of reduced training and training cessation on insulin action and muscle GLUT-4. Journal of Applied Physiology 1996;81:1162-1168.

97. Liang KY, Mintun MA, Fagan AM, et al. Exercise and Alzheimer's disease biomarkers in cognitively normal older adults. Ann Neurol 2010;68:311-318.

98. Head D, Bugg JM, Goate AM, et al. Exercise Engagement as a Moderator of the Effects of APOE Genotype on Amyloid Deposition. Arch Neurol:archneurol.2011.2845.

99. Deeny SP, Poeppel D, Zimmerman JB, et al. Exercise, APOE, and working memory: MEG and behavioral evidence for benefit of exercise in epsilon4 carriers. Biol Psychol 2008;78:179-187.

100. Etnier JL, Caselli RJ, Reiman EM, et al. Cognitive performance in older women relative to ApoE-epsilon4 genotype and aerobic fitness. Med Sci Sports Exerc 2007;39:199-207.

101. Kivipelto M, Rovio S, Ngandu T, et al. Apolipoprotein E epsilon4 Magnifies Lifestyle Risks for Dementia: A Population Based Study. J Cell Mol Med 2008.

102. Niti M, Yap KB, Kua EH, Tan CH, Ng TP. Physical, social and productive leisure activities, cognitive decline and interaction with APOE-epsilon 4 genotype in Chinese older adults. Int Psychogeriatr 2008;20:237-251.

103. Rovio S, Kareholt I, Helkala EL, et al. Leisure-time physical activity at midlife and the risk of dementia and Alzheimer's disease. Lancet Neurol 2005;4:705-711.

104. Schuit AJ, Feskens EJ, Launer LJ, Kromhout D. Physical activity and cognitive decline, the role of the apolipoprotein e4 allele. Med Sci Sports Exerc 2001;33:772-777.

105. Podewils LJ, Guallar E, Kuller LH, et al. Physical activity, APOE genotype, and dementia risk: findings from the Cardiovascular Health Cognition Study. Am J Epidemiol 2005;161:639-651.

106. Lindsay J, Laurin D, Verreault R, et al. Risk Factors for Alzheimerâ€™s Disease: A Prospective Analysis from the Canadian Study of Health and Aging. American Journal of Epidemiology 2002;156:445-453.

107. Sabia S, Kivimaki M, Kumari M, Shipley MJ, Singh-Manoux A. Effect of Apolipoprotein E epsilon4 on the association between health behaviors and cognitive function in late midlife. Mol Neurodegener;5:23.

108. Taaffe DR, Irie F, Masaki KH, et al. Physical Activity, Physical Function, and Incident Dementia in Elderly Men: The Honoluluâ€“Asia Aging Study. The Journals of Gerontology Series A: Biological Sciences and Medical Sciences 2008;63:529-535.

109. Burns JM, Honea RA, Vidoni ED, Hutfles LJ, Brooks WM, Swerdlow RH. Insulin is differentially related to cognitive decline and atrophy in Alzheimer's disease and aging. Biochimica et Biophysica Acta (BBA) - Molecular Basis of Disease 2012;1822:333-339.

110. Honea RA, Swerdlow RH, Vidoni ED, Burns JM. Progressive regional atrophy in normal adults with a maternal history of Alzheimer disease. Neurology 2011;76:822-829.

111. Daviglus ML, Bell CC, Berrettini W, et al. NIH State-of-the-Science Conference Statement: Preventing Alzheimer's Disease and Cognitive Decline. NIH Consens State Sci Statements 2010;27.

112. Morris JC. The Clinical Dementia Rating (CDR): current version and scoring rules. Neurology 1993;43:2412b-2414.

113. Hughes CP, Berg L, Danziger WL, Coben LA, Martin RL. A new clinical scale for the staging of dementia. British Journal of Psychiatry 1982;140:566-572.

114. Mayer CJ, Steinman L, Williams B, Topolski TD, LoGerfo J. Developing a Telephone Assessment of Physical Activity (TAPA) questionnaire for older adults. Prev Chronic Dis 2008;5:A24.

115. ACSM ACoSM. ACSM's guidelines for exercise testing and prescription, 8th ed. Philladelphia: Lippincott Williams & Wilkins, 2010.

116. Morss GM, Jordan AN, Skinner JS, et al. Dose Response to Exercise in Women aged 45-75 yr (DREW): design and rationale. Med Sci Sports Exerc 2004;36:336-344.

117. Voss MW, Prakash RS, Erickson KI, et al. Plasticity of brain networks in a randomized intervention trial of exercise training in older adults. Front Aging Neurosci 2010;2.

118. Colcombe SJ, Erickson KI, Scalf PE, et al. Aerobic exercise training increases brain volume in aging humans. J Gerontol A Biol Sci Med Sci 2006;61:1166-1170.

119. Rinne JO, Brooks DJ, Rossor MN, et al. 11C-PiB PET assessment of change in fibrillar amyloid-? load in patients with Alzheimer's disease treated with bapineuzumab: a phase 2, double-blind, placebo-controlled, ascending-dose study. The Lancet Neurology;9:363-372.

120. Vlassenko AG, Mintun MA, Xiong C, et al. Amyloid-beta plaque growth in cognitively normal adults: Longitudinal [11C]Pittsburgh compound B data. Annals of Neurology 2011;70:857-861.

121. Jack CR, Jr., Slomkowski M, Gracon S, et al. MRI as a biomarker of disease progression in a therapeutic trial of milameline for AD. Neurology 2003;60:253-260.

122. Squire LR. Memory and the hippocampus: a synthesis from findings with rats, monkeys, and humans. Psychol Rev 1992;99:195-231.

123. Braak H, Braak E. Neuropathological stageing of Alzheimer-related changes. Acta Neuropathol (Berl) 1991;82:239-259.

124. Ikonomovic MD, Klunk WE, Abrahamson EE, et al. Precuneus amyloid burden is associated with reduced cholinergic activity in Alzheimer disease. Neurology 2011;77:39-47.

125. Buckner RL, Snyder AZ, Shannon BJ, et al. Molecular, Structural, and Functional Characterization of Alzheimer's Disease: Evidence for a Relationship between Default Activity, Amyloid, and Memory. J Neurosci 2005;25:7709-7717.

126. Karas G, Scheltens P, Rombouts S, et al. Precuneus atrophy in early-onset Alzheimer's disease: a morphometric structural MRI study. Neuroradiology 2007;49:967-976.

127. Wolk DA, Price JC, Saxton JA, et al. Amyloid imaging in mild cognitive impairment subtypes. Ann Neurol 2009;65:557-568.

128. Drzezga A, Becker JA, Van Dijk KR, et al. Neuronal dysfunction and disconnection of cortical hubs in non-demented subjects with elevated amyloid burden. Brain 2011;134:1635-1646.

129. Erickson KI, Raji CA, Lopez OL, et al. Physical activity predicts gray matter volume in late adulthood: The Cardiovascular Health Study. Neurology 2010;75:1415-1422.

130. Johnson DK, Barrow W, Anderson R, et al. Diagnostic utility of cerebral white matter integrity in early Alzheimer's disease. The International journal of neuroscience 2010;120:544-550.

131. Dale AM, Fischl B, Sereno MI. Cortical Surface-Based Analysis: I. Segmentation and Surface Reconstruction. Neuroimage 1999;9:179-194.

132. Reuter M, Fischl B. Avoiding asymmetry-induced bias in longitudinal image processing. Neuroimage 2011.

133. Honea RA, Vidoni E, Harsha A, Burns JM. Impact of APOE on the Healthy Aging Brain: A Voxel-Based MRI and DTI Study. J Alzheimers Dis 2009;18:553-564.

134. Vidoni ED, Honea RA, Burns JM. Neural correlates of impaired functional independence in early Alzheimer's disease. J Alzheimers Dis 2010;19:517-527.

135. Dustman RE, Emmerson RY, Ruhling RO, et al. Age and fitness effects on EEG, ERPs, visual sensitivity, and cognition. Neurobiology of aging 1990;11:193-200.

136. Ewers M, Insel P, Jagust WJ, et al. CSF Biomarker and PIB-PET-Derived Beta-Amyloid Signature Predicts Metabolic, Gray Matter, and Cognitive Changes in Nondemented Subjects. Cereb Cortex 2011.

137. Buchhave P, Janciauskiene S, Zetterberg H, Blennow K, Minthon L, Hansson O. Elevated plasma levels of soluble CD40 in incipient Alzheimer's disease. Neurosci Lett 2009;450:56-59.

138. Buchhave P, Stomrud E, Warkentin S, Blennow K, Minthon L, Hansson O. Cube copying test in combination with rCBF or CSF A beta 42 predicts development of Alzheimer's disease. Dementia and geriatric cognitive disorders 2008;25:544-552.

139. Storandt M, Mintun MA, Head D, Morris JC. Cognitive decline and brain volume loss as signatures of cerebral amyloid-beta peptide deposition identified with Pittsburgh compound B: cognitive decline associated with Abeta deposition. Archives of neurology 2009;66:1476-1481.

140. Mormino EC, Kluth JT, Madison CM, et al. Episodic memory loss is related to hippocampal-mediated beta-amyloid deposition in elderly subjects. Brain : a journal of neurology 2009;132:1310-1323.

141. Rentz DM, Locascio JJ, Becker JA, et al. Cognition, reserve, and amyloid deposition in normal aging. Annals of neurology 2010;67:353-364.

142. Johnson DK, Storandt M, Morris JC, Galvin JE. Longitudinal study of the transition from healthy aging to Alzheimer disease. Archives of neurology 2009;66:1254-1259.

143. Wilson RS, Leurgans SE, Boyle PA, Bennett DA. Cognitive decline in prodromal Alzheimer disease and mild cognitive impairment. Archives of neurology 2011;68:351-356.

144. Mikels JA, Larkin GR, Reuter-Lorenz PA, Cartensen LL. Divergent trajectories in the aging mind: changes in working memory for affective versus visual information with age. Psychology and aging 2005;20:542-553.

145. Amieva H, Letenneur L, Dartigues JF, et al. Annual rate and predictors of conversion to dementia in subjects presenting mild cognitive impairment criteria defined accordign to a population-based study. DementGeriatrCognDisord 2004;18:87-93.

146. Miyake A, Friedman NP, Rettinger DA, Shah P, Hegarty M. How are visuospatial working memory, executive functioning, and spatial abilities related? A latent-variable analysis. J Exp Psychol Gen 2001;130:621-640.

147. Erickson KI, Voss MW, Prakash RS, et al. Exercise training increases size of hippocampus and improves memory. Proc Natl Acad Sci U S A 2011;108:3017-3022.

148. Thurstone LL, Thurstone LG. Examiner Manual for the SRA Primary Mental Abilities Test. Chicago: Science Research Associates, 1949.

149. Goodglass H, Kaplan E. Boston Naming Test scoring booklet. Philadelphia: Lea & Febiger, 1983.

150. Wechsler D. Adminstration and scoring manual: Wechsler Adult Intelligence Scale. San Antonio, TX: Harcourt Brace, 1997.

151. Wechsler D. Wechsler Memory Scale III: Administration and scoring manual. Orlando, FL: Psychological Corporation, 1997.

152. Stroop J. Studies of interference in serial verbal reactions. J Exp Psychol 1935;18:643-662.

153. Armitage SG. An analysis of certain psychological tests used for the evaluation of brain injury. Psychological Monographs 1946;60:277.

154. Delis DC, Kaplan E, Kramer JH. Delis–Kaplan Executive Function System (D-KEFS). San Antonio: Pearson Education Incorporated, 2001.

155. Grober E, Buschke H, Crystal H, Bang S, Dresner R. Screening for dementia by memory testing. Neurology 1988;38:900-903.

156. Wechsler DA. Manual: Wechsler Adult Intelligence Scale - Fourth Edition. New York: The Psychological Corporation, 2008.

157. Johnson DK, Morris JC, Galvin JE. Verbal and visuospatial deficits in dementia with Lewy bodies. Neurology 2005;65:1232-1238.

158. Johnson DK, Storandt M, Morris JC, Langford ZD, Galvin JE. Cognitive profiles in dementia: Alzheimer disease vs nondemented aging. Neurology 2008;71:1783-1789.

159. Salthouse TA, Ferrer-Caja E. What needs to be explained to account for age-related effects on multiple cognitive variables? Psychology and aging 2003;18:91-110.

160. Zelinski EM, Lewis KL. Adult age differences in multiple cognitive functions: differentiation, dedifferentiation, or process-specific change? Psychology and aging 2003;18:727-745.

161. Johnson DK, Galvin JE. Longitudinal changes in cognition deficits in Parkinson’s disease with and without dementia. Dementia and geriatric cognitive disorders 2011;31:98-108.

162. Pretz JE, Sternberg RJ. Unifying the field: Cognition and intelligence. Book; Edited Book; Chapter, 2005 306-318). New York, NY, US: Cambridge University.

163. Cole DA, Maxwell SE. Testing mediational models with longitudinal data: questions and tips in the use of structural equation modeling. J Abnorm Psychol 2003;112:558-577.

164. Burns JM, Mayo MS, Anderson HS, Smith H, Donnelly JE. Cardiorespiratory Fitness in Early-Stage Alzheimer’s Disease. . Alzheimer Dis Assoc Disord 2008;22:39-46.

165. Hollenberg M, Ngo LH, Turner D, Tager IB. Treadmill Exercise Testing in an Epidepiologic Study of Elderly Subjects. Journal of Gerontology: Biological Sciences 1998;53A:259-267.

166. Hacker M, Stork S, Stratakis D, et al. Relationship between right ventricular ejection fraction and maximum exercise oxygen consumption: a methodological study in chronic heart failure patients*1. Journal of Nuclear Cardiology 2003;10:644-649.

167. Fleg JL, Morrell, C. H., Bos, A. G., et al. Accelerated Longitudinal Decline of Aerobic Capacity in Healthy Older Adults. Circulation 2005;112:674-682.

168. Duncan GE, Howley ET, Johnson BN. Applicability of VO2max criteria: discontinuous versus continuous protocols. Medicine And Science In Sports And Exercise 1997;29:273-278.

169. Kohrt WM. Preliminary evidence that DEXA provides an accurate assessment of body composition. Journal of Applied Physiology 1998;84:372-377.

170. Pintauro SJ, Nagy TR, Duthie CM, Goran MI. Cross-calibration of fat and lean measurements by dual-energy X-ray absorptiometry to pig carcass analysis in the pediatric body weight range. American Journal of Clinical Nutrition 1996;63:293-298.

171. Shah KR, Carr D, Roe CM, Miller JP, Coats M, Morris JC. Impaired Physical Performance and the Assessment of Dementia of the Alzheimer Type. Alzheimer Disease and Associated Disorders 2004;18:112-118.

172. Villareal DT, Banks M, Sinacore DR, Siener C, Klein S. Effect of Weight Loss and Exercise on Frailty in Obese Older Adults. Arch Intern Med 2006;166:860-866.

173. Rikli R, Jones J. Development and validation of a functional fitness test for community-residing older adults. J Aging Phys Act 1999;7:129-161.

174. Davidson LE, Hudson R, Kilpatrick K, et al. Effects of exercise modality on insulin resistance and functional limitation in older adults: a randomized controlled trial. Arch Intern Med 2009;169:122-131.

175. Al Snih S, Markides KS, Ottenbacher KJ, Raji MA. Hand grip strength and incident ADL disability in elderly Mexican Americans over a seven-year period. Aging Clin Exp Res 2004;16:481-486.

176. Jette AM, Haley SM, Coster WJ, et al. Late life function and disability instrument: I. Development and evaluation of the disability component. J Gerontol A Biol Sci Med Sci 2002;57:M209-216.

177. Haley SM, Jette AM, Coster WJ, et al. Late Life Function and Disability Instrument: II. Development and evaluation of the function component. J Gerontol A Biol Sci Med Sci 2002;57:M217-222.

178. Ouellette MM, LeBrasseur NK, Bean JF, et al. High-intensity resistance training improves muscle strength, self-reported function, and disability in long-term stroke survivors. Stroke; a journal of cerebral circulation 2004;35:1404-1409.

179. Morey MC, Snyder DC, Sloane R, et al. Effects of home-based diet and exercise on functional outcomes among older, overweight long-term cancer survivors: RENEW: a randomized controlled trial. JAMA : the journal of the American Medical Association 2009;301:1883-1891.

180. Harris PA, Taylor R, Thielke R, Payne J, Gonzalez N, Conde JG. Research electronic data capture (REDCap)--a metadata-driven methodology and workflow process for providing translational research informatics support. J Biomed Inform 2009;42:377-381.
