# Supplementary material for: Effect of aerobic exercise on amyloid accumulation in preclinical Alzheimer’s: A 1-year randomized controlled trial
Source: PLoS One. 2021 Jan 14;16(1):e0244893. doi: 10.1371/journal.pone.0244893 (PMC7808620; doi:10.1371/journal.pone.0244893)
Supplement: Attachment — Submitted filename: APEX Primary R2R 12.15.2020.docx [file pone.0244893.s006.docx]

Dr. Bush,

Academic Editor, PLOS One

We appreciate the final reviews of our manuscript PONE-D-20-24208R1, “*Effect of Aerobic Exercise on Amyloid Accumulation in Preclinical Alzheimer’s: A 1-Year Randomized Controlled Trial*”. We have made the final requested correction

In the analysis section our sentence now reads:

*“Descriptive statistics were generated, including means, standard deviations and ranges for continuous measures, and frequencies and relative frequencies for categorical measures.”*

We have provided a “clean” copy of our revised manuscript. We thank the reviewers for helping us to improve the manuscript and look forward to positive reception.

Sincerely,

Jeffrey M. Burns, MD, MS

University of Kansas Medical Center.
